# Open Hardware in Science: The Benefits of Open Electronics

**DOI:** 10.1093/icb/icac043

**Published:** 2022-05-20

**Authors:** Michael Oellermann, Jolle W Jolles, Diego Ortiz, Rui Seabra, Tobias Wenzel, Hannah Wilson, Richelle L Tanner

**Affiliations:** Aquatic Systems Biology Unit, TUM School of Life Sciences, Technical University of Munich, Mühlenweg 22, D-85354 Freising, Germany; Institute for Marine and Antarctic Studies, Fisheries and Aquaculture Centre, University of Tasmania, Private Bag 49, Hobart, TAS 7001, Australia; Centre for Research on Ecology and Forestry Applications—CREAF, Campus UAB, Edifici C. 08193, Bellaterra Barcelona, Spain; Instituto Nacional de Tecnología Agropecuaria—INTA, Estación Experimental Manfredi, Ruta 9, Km 636, 5988 Manfredi, Córdoba, Argentina; CIBIO, Centro de Investigação em Biodiversidade e Recursos Genéticos, InBIO Laboratório Associado, Campus de Vairão, Universidade do Porto, 4485-661 Vairão, Portugal; BIOPOLIS Program in Genomics, Biodiversity and Land Planning, CIBIO, Campus de Vairão, 4485-661 Vairão, Portugal; Institute for Biological and Medical Engineering, Schools of Engineering, Medicine and Biological Sciences, Pontificia Universidad Católica de Chile, Vicuña Mackenna 4860, Macul 7820244, Santiago, G92Q+26, Chile; Biology Department, College of Science, Utah State University, 5305 Old Main Hill, Logan, UT 84321, USA; Environmental Science and Policy Program, Chapman University, 1 University Drive, Orange, CA 92866, USA

## Abstract

Openly shared low-cost electronic hardware applications, known as open electronics, have sparked a new open-source movement, with much untapped potential to advance scientific research. Initially designed to appeal to electronic hobbyists, open electronics have formed a global “maker” community and are increasingly used in science and industry. In this perspective article, we review the current costs and benefits of open electronics for use in scientific research ranging from the experimental to the theoretical sciences. We discuss how user-made electronic applications can help (I) individual researchers, by increasing the customization, efficiency, and scalability of experiments, while improving data quantity and quality; (II) scientific institutions, by improving access to customizable high-end technologies, sustainability, visibility, and interdisciplinary collaboration potential; and (III) the scientific community, by improving transparency and reproducibility, helping decouple research capacity from funding, increasing innovation, and improving collaboration potential among researchers and the public. We further discuss how current barriers like poor awareness, knowledge access, and time investments can be resolved by increased documentation and collaboration, and provide guidelines for academics to enter this emerging field. We highlight that open electronics are a promising and powerful tool to help scientific research to become more innovative and reproducible and offer a key practical solution to improve democratic access to science.

## Introduction

The revolutionary open science movement has helped to foster transparency, collaborative access, and sharing of scientific knowledge (Vicente-Saez and Martinez-Fuentes 2018). Open science started with open-access publications and has now expanded to liberate access to data, programming code, and even lab notebooks (Boulton et al. 2012; McCray et al. 2018; Vicente-Saez and Martinez-Fuentes 2018). However, so far one domain that is at the very core of scientific data production has been less prominent in the open science movement: hardware, electronics, and instruments (Harnett 2011; Pearce 2012; Maia Chagas 2018). Proprietary instruments support high-profile research, such as microfluidic instruments for transcriptomics or autonomous underwater vehicles to monitor marine environments, yet high costs limit their access only to well-funded labs. Most researchers globally do not have access to the funding required to buy state-of-the-art proprietary instruments, limiting both reproducibility and innovation potential (van Helden 2012). Free and open-source hardware (Pearce 2013) has the potential to close this divide: in many instances, it can match high-end performance of proprietary instrumentation while facilitating the sharing of free-of-cost design blueprints to re-build, modify, or advance instruments, and fostering collaboration with other scientists and a worldwide community of “makers,” civic scientists, and hobbyist inventors (Pearce 2012; Maia Chagas 2018).

**Figure 1.**
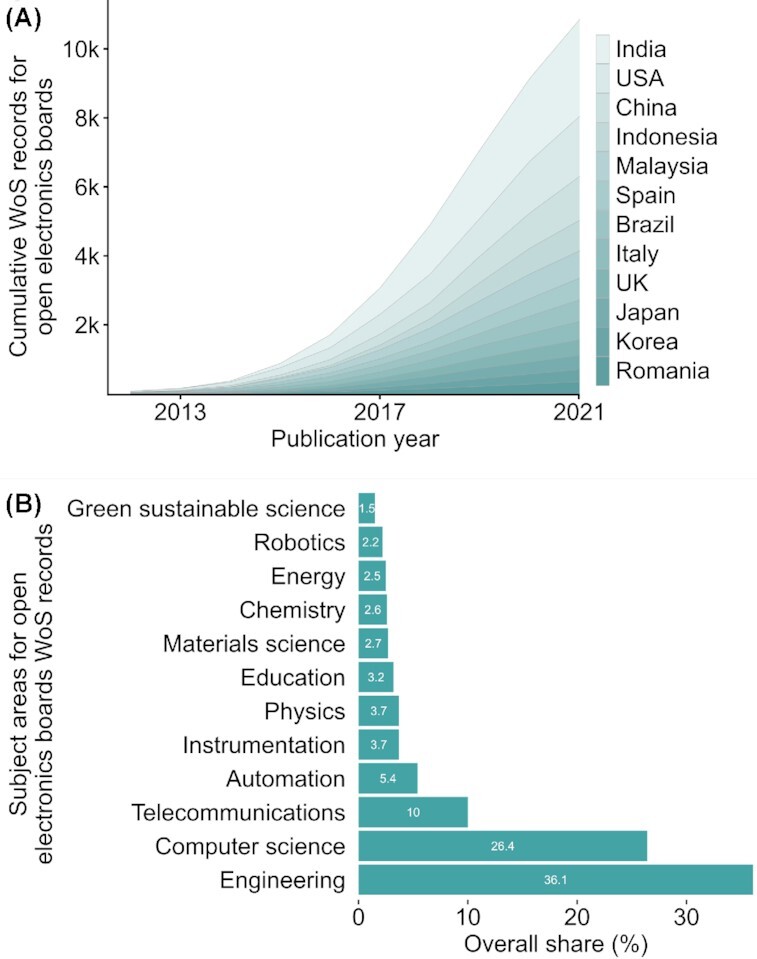
**(A)** Cumulative growth of Web of Science records grouped by the top 12 countries and **(B)** dominant subject areas. The search comprised the open-electronics boards listed in Fig. 3 and included articles and proceeding papers between 2010 and 2020 and the country origins for authors and co-authors. For detailed analysis, bibliographic data, country distribution, and separate analysis for proceedings articles only, see Supplementary Information File S1.

Electronics are a major component of the open hardware domain, which provides open-design scientific hardware solutions (Pearce 2012; Bonvoisin et al. 2020). Such solutions are often built in combination or solely with particular electronic hardware components whose main purpose is to allow non-experts to easily create or reproduce electronic applications. Therefore, in contrast to conventional electronics, we define “open electronics” as applications and designs that are assembled from very accessible components and openly shared to facilitate access, learning, and reproducibility at minimized costs. This contrasts with commercial applications, and proprietary or closed designs, which restrict access and use of designs by license ownership. To clarify, many open electronics rely on components that are not open-source and may in fact may be closed-design (e.g., the Raspberry Pi [Raspberry Pi Foundation, Cambridge, United Kingdom]) or commercial (e.g., the DS18B20 sensor [Maxim Integrated, San Jose, CA, USA]), but they are well-suited to create affordable, accessible, and broad electronics applications. Applications and their designs should be openly published and shared, which, depending on their novelty and complexity, can range from a short description in the methods or supplementary material section of a paper; online repositories (e.g., open-neuroscience.com, Maia Chagas 2021); detailed step-by-step instructions in methods articles or websites (e.g., Geissmann et al. 2017); to publications entirely focused on describing tool development and application potential (Plum and Labonte 2021). Components dedicated to the open-electronics concept include single-board microcomputers and micro-controllers that can be easily interfaced with a plethora of hardware modules, sensors, and actuators (Table 1), and often without much technical experience or highly specialized tools. In combination with the modular nature of many open-electronics platforms, users do not need to invent applications from scratch and can gradually grow skills and application complexity. By only requiring basic programming and electronics skills in most instances, detailed documentation and components being commonly available, and with help of numerous open online tutorials and databases (e.g., instructables.com; hackster.io), open-electronics projects have become very accessible to the broader public. With millions of hobbyist “makers” and DIYers around the globe—for example, already >37 million Raspberry Pi microcomputers have been sold since its release in 2012 (RaspberryPiFoundation 2020)—the popularity of open electronics has continued to rise and is beginning to establish in diverse scientific domains, including the biological sciences (Fig. 1A and B; Jolles 2021a).

**Table 1.** Overview of the wide range of sensors and actuators available that are compatible with open-electronics boards and platforms

Sensors	
Environment	Temperature, humidity, barometric pressure, soil moisture, particulate matter, light intensity, smoke, dust, radiation, sound
Water	Chlorine, pH, depth and pressure, liquid level, flow, turbidity
Gas	CO, CO_2_, alcohol, H_2_, TVOCs, ozone, H_2_S, CH_4_, NO
Movement	Distance, acceleration, seismic, GPS, break-beam, motion
Biometrics	Heart rate, muscle activity, fingerprints, weight/load, force
Imaging	Spectroscopy, visible and IR range cameras, thermal imaging, gestures
Other	Magnetism, capacitive touch, current, voltage, RFID, PIT tags
Actuators	
Light	LEDs, infrared, UV, laser
Movement	Servos, stepper motor, gear motor, vacuum pumps, valves
Switches	Mechanical, electrical, magnetic, DC and AC relays
Other	Vibration, sound, ultrasound, Peltier heating/cooling

Despite their increasing uptake in science, open-electronics applications are far from being widespread. Poor awareness, inadequate documentation, and insufficient electronic literacy outside the engineering and computer sciences have contributed to its fragmented and uneven use across scientific subjects (Fig. 1B). In comparison, an open-source software project such as the R statistical language (R Core Team 2021) had similar challenges at the start but has now become one of the most popular data tools in science (Muenchen 2012; Lai et al. 2019) due to its high flexibility and rapid adaptation to new research demands and trends via user-driven innovation networks (Von Hippel 2005, 2007). To reach broad-scale uptake of open electronics, important barriers need to be overcome by increasing access to detailed information, improving educational and research support, and by presenting a clear case for how open electronics can benefit researchers, institutions, and the scientific community alike. Increased awareness and use across the biological sciences will advance discussions to more complex barriers, such as constrained funding or resources, user-centered design, or remote location challenges. Open electronics can aid solving some of those barriers with their low cost, easy to build designs, and better global accessibility. Such actions will help to adopt open electronics more broadly and accelerate technical innovation, lower research costs, enable highly customizable solutions, and democratize hardware access in experimental science (Pearce 2015, 2016).

In this paper, we outline the broad benefits that open electronics can have from the level of the researcher to that of institutions and the scientific community at large. We also discuss current barriers and trade-offs, provide a “beginner's toolbox” to help researchers get started, and conclude with an outlook discussing potential impacts on science and required actions for a broad uptake. Overall, it is not our aim to persuade researchers to use open electronics in their work but to provide a comprehensive account and raise awareness of their multi-level benefits as a very accessible technological solution for experimental inquiries, as we believe open electronics have great potential to enhance the innovation, reproducibility, and democratization of science.

## Application potential for open electronics

Open electronics offer a versatile spectrum of applications to a wide range of users in science, education, and industry, besides the general public. Although initially used only by the most electronics-savvy hobbyists and Do-it-Yourself creators, open electronics are increasingly taken up by broader public audiences spanning all age groups, further fueled by the rise of the Internet of Things (Ibrahim et al. 2015). Automation of scheduled tasks such as watering plants in the garden (Divani et al. 2016) or controlling household devices (i.e., smart homes) are very popular and easy to set up (Hasan et al. 2018). This extends to various measurements and surveillance applications, such as automated weather stations and nest box systems, and even the development of smart cities (Costa and Duran-Faundez 2018). Some of the driving forces behind the rise of open electronics are the aim to make computing and electronics accessible to anyone, such as in STEM education, and to introduce students to electronics and programming basics as well as solving practical problems and practicing the scientific method (discussed in Jolles 2021a). The increasing interest in open electronics as teaching tools is supported by an extensive pool of learning resources for teaching and self-learning (see Table 2). This also supports scientists to try out and test new ideas, learn new approaches, or develop own customized applications on a small budget.

**Table 2.** Collection of online resources and communities, for both beginners and advanced users, with hyperlinks

Resource	Link	Description
Arduino website	arduino.cc	Many tutorials, forums, blog posts and products for sale
Biomaker website	biomaker.org	No-code programming tutorials for biologists
Raspberry Pi website	projects.raspberrypi.org	Many tutorials, forums, blog posts and products for sale
Raspberry Pi Beginner's Guide	magpi.raspberrypi.org/books/beginners-guide-4th-ed	Free guidebook for getting started with Raspberry Pi
Adafruit website	learn.adafruit.com	Thorough documentation and tutorials for Adafruit products
Sparkfun website	learn.sparkfun.com	Thorough documentation and tutorials for Sparkfun products
PiHut website	thepihut.com/blogs	Thorough documentation and tutorials for PiHut products
Raspberry Pi Guide	raspberrypi-guide.github.io	A collection of 30 + Raspberry Pi tutorials specifically written for scientists ([Bibr bib38][Bibr bib38])
Coursera	Coursera.org	Offers courses on topics related to electronics and computing
Udemy	Udemy.com	Offers courses on topics related to electronics and computing
TinkerCad	tinkercad.com	Lets you build virtual versions of circuits to test your wiring and code
Raspberry Pi Forums	raspberrypi.org/forums	300k + member forum to ask questions about Raspberry Pi
Open Science Hardware Forum	forum.openhardware.science	Forum to ask questions related to open hardware and global community events
Stack Overflow	stackoverflow.com	Forum to ask questions about hardware or coding
Open-Neuroscience	open-neuroscience.com	Database for scientific open-hardware designs
Github	Githubpages	Site to create a free, version controlled online website with your documentation
Wildlabs.net	wildlabs.net	Conservation technology network
Conservation X Labs	conservationxlabs.com	Technology and innovation company working against extinction
Open Source Ecology	opensourceecology.org	Ecology-relevant solutions and community around an open-source economy

Many companies that sell components for open electronics provide thorough documentation and tutorials. Furthermore, there are guides specifically developed for scientists wanting to work with the Raspberry Pi or Arduino (e.g., Jolles 2021b ), and an increasing number of online courses are available on topics related to electronics and computing. The links above are arranged by relevance—starting with beginner tutorials and ending with ways to share applications.

So far, despite much potential, there has only been a marginal uptake of open electronics in biological research and science generally, with predominant use in the engineering and computer sciences (Fig. 1B). However, also in the biological sciences, open-electronics applications have increased in number over the last few years, with numerous solutions found both in traditional journals as well as newly established open hardware journals (e.g., Journal of Open Hardware , HardwareX).

Some examples from the field of behavioral ecology include video recording, tracking, and remote solutions, such as the high-throughput automated recording of the behavior of individuals and groups of fish (Jolles et al. 2017); *in-situ* analysis of zooplankton phototactic behavior underwater (Lertvilai and Durand 2020); ”biologging” of migratory and nesting birds (Bridge et al. 2019; Youngblood 2020); and the analysis of kangaroo rat escape behavior (Schwaner et al. 2021).

At the ecosystem-scale, solar-powered Raspberry Pis, cameras, and microphones have enabled the fully autonomous acoustic and visual monitoring of ecosystems with remote data transmission for <$320 USD (Sethi et al. 2018), and the long-term visual, high-frequency monitoring of forest canopy cover (Wilkinson et al. 2021). Experimental biologists tested the impact of light spectral quality and intensity on plant host–parasite interaction using an Arduino-controlled LED array (Johnson et al. 2016) (Arduino, Somerville, MA, SUS), and soil ecologists developed a “smart electronics nose” to monitor soil organic richness using Arduino-read gas sensors and IoT ZigBee boards to stream data from the field to the lab (Dorji et al. 2017).

In evolutionary biology, open electronics were used to perform high-throughput experiments to automatically simulate and identify adaptive niches of yeast (Wong et al. 2018). These examples impressively demonstrate the versatile and fast-growing application potential of open electronics in biological research. Besides numerous applications in other scientific disciplines, they are also formidable tools for community science and scientific outreach activities, such as school student-operated ocean observers (Beckler et al. 2018), urban air pollution monitors (Jiang et al. 2016), interactive autonomously moving lights to teach about animal behavior (Jolles 2021, pers. comm.), and sonic kayaks to monitor underwater soundscapes (Griffiths et al. 2017).

## Research benefits of open electronics

In addition to their diverse application potential, open electronics can provide a broad range of significant benefits at the different levels of academia and resolve important practical, financial, and structural issues in science. Below we discuss the key benefits for individual researchers, for research institutions, and for the academic community at large.

### Benefits to individual researchers

#### Wide applicability, from simple to comple*x*

Unlike most commercial scientific instruments, which confine customers and applications to product lines, open electronics are highly flexible and adaptable and can be implemented in a broad range of applications, from basic to the highly complex, such as simple video recording of snakes (Zamore et al. 2020), advanced IoT-based bird monitoring nest boxes (Abdelouahid et al. 2020), or complex real-time virtual reality systems to study chemotaxis in flies (Tadres and Louis 2020). Users can start simple and expand their devices with increasing programming and electronics skills, like starting with only logging lab temperature, then displaying it live on an LCD screen, controlling heaters to regulate temperature to, finally, a complete stand-alone system with multiple sensors, warning messages, and interactive graphical user interfaces (GUIs). Users can also easily repurpose open electronics by reusing components from previous setups for new or more complex builds.

#### Broad sensor and actuator application potential

A major strength of open electronics is the wide range of sensors and actuators available that can be controlled with the accuracy of reference equipment (Table 1; Setyowati et al. 2017). Open electronics can also be used in applications requiring an especially small size (Shipley et al. 2017), both in the lab and under harsh conditions in the field (e.g., Beddows and Mallon 2018). Micro-controllers and single-board computers also enable multiple sensors and actuators to be connected simultaneously, providing a sensing and reactive capacity that, in many instances, can outperform complete proprietary solutions while having significantly lower individual hardware unit needs, costs, and power consumption.

#### Experimental automation

Repetitive tasks, such as control and recording of experimental parameters in the field or lab, animal feeding, and monitoring of experimental trials, are amongst the most time-consuming factors in research. Open electronics can benefit researchers by automating such tasks, including by using pipetting robotics in eco-toxicological assays (Steffens et al. 2017), RFID-based animal feeding stations (Bridge et al. 2019), or acoustic sensor networks generating high-density data streams from the field to the cloud (Roe et al. 2018). Task automation also helps reduce human error and experimental variability (Eggert et al. 2020) and increases resilience to unforeseen circumstances, by using automated monitoring systems to alert researchers when equipment fails or experimental conditions change unexpectedly (Gurdita et al. 2016).

#### Customization

Most instruments, such as hand-held meters, data loggers, and PCR machines, are closed entities, constrained to the functions set by the manufacturer and operating software, and can thus become redundant if research needs change. The poor ability to modify or expand functionalities also confines the scope and implementation of new research ideas. Open electronics provides one solution, as researchers can not only develop or retrofit existing open-electronics setups and devices, exchange, or program new operations, but also link and expand the features of existing laboratory instruments. For example, micro-controllers and single-board computers can interface with proprietary instruments via serial ports and hardware communication protocols, to query information or execute functions, while adding new functionalities using sensors and actuators (Stewart et al. 2017; Virag et al. 2021). As many boards offer wireless connectivity with an increasing focus on Internet-of-Things applications (e.g., NodeMCU ESP32 [Espressif Systems, Shanghai, China], Fig. 3), even simple weighing scales can integrate into a smart instrument network, channeling and summarizing data streams in cloud-based dashboards (Poongothai et al. 2018; Arunachalam and Andreasson 2021). Microscopes have especially benefited from this customizability, leading to many high-specification or low-cost open platforms (Hohlbein et al. 2021).

#### Scalability and high throughput

Open electronics provide researchers with the opportunity to easily scale and replicate setups to suit singular or high-throughput applications. Their low cost and off-the-shelf availability enables quick and low-risk prototyping up until a well-functioning setup that can be copied to create whole arrays of identical devices, such as to GPS-track tens of animals (Foley and Sillero‐Zubiri 2020), to test the behavior of hundreds of individual flies (Geissmann et al. 2017), to observe the growth of thousands of plants (Tausen et al. 2020), and to simulate multi-dimensional environmental conditions by parallelized automated processing of sample microvolumes (Wong et al. 2018). Such scalability is particularly valuable when funding is limited, enabling researchers to begin with simpler setups, rather than facing high upfront costs for proprietary systems.

#### Flexible data access and programming capabilities

Open electronics are highly flexible in terms of data acquisition, formats, storage, and accessibility. Numerous libraries in a broad range of programming languages make it possible to read sensor data in a few lines of code. Library-rich easy-to-read programming languages such as Python, further facilitate endless possibilities to work with custom electronics and devices, including automatic data processing actions such as folder monitoring (e.g., watchdog library), file conversion, and automatic creation of data backups (e.g., BackuPy library). Data can also be accessed remotely, including from a local network and the Internet, and from remote field locations via mobile network adaptors (e.g., RPyC library, Sethi et al. 2018). This, in turn, enables the continuous real-time remote monitoring of data, such as of lab conditions, animal activity, plant growth, and environmental variables in the field (Jolles 2020; Siregar et al. 2017; Trasviña-Moreno et al. 2017). Improved computing power of single-board computers has made it increasingly possible to process data onboard, enabling the transmission of flagged or summarized data for researchers (Allan et al. 2018). Data can also be visualized in a professional manner via custom-build user interfaces or online dashboards, supported by numerous graphical libraries, many of which are open-source (e.g., Tkinter, PyQT, WxPython, Dash, and Plotly) (Boudoire et al. 2020; Lewinski et al. 2020).

#### Simple maintenance

Most components of open electronics can be easily serviced and replaced by the users themselves, with most parts likely available at online retailers and electronic hardware stores. Also, required tools, such as soldering equipment and a multi-meter, tend to be highly affordable. In contrast, when issues occur with proprietary (scientific) instruments, custom repairs, even when feasible, are not recommended as they break product warranty. Researchers therefore rely on manufacturers for repairs, which can be time-consuming and expensive, specifically for regions distant from industrial and trade hubs, where return shipments or service staff visits are prohibitively costly. Product support may also cease if products become outdated or companies stop existing. Therefore, open electronics aid researchers to depend less on exclusive vendors’ support and be more resilient against potential technical problems.

#### Extensive learning resources and community support

Extensive learning resources, including a large range of books and free tutorial websites (see Table 2), and an increasing number of open online courses (e.g., Coursera and Udemy) offer many ways to learn about open electronics and how to build custom applications. Academic papers now often come with supplementary guides and accompanying websites about methodologies (e.g., Geissmann et al. 2017; Maia Chagas 2021), and several specialized journals exist to help researchers build and publish their own devices (e.g., Journal of Open Hardware (Murillo and Wenzel 2017) and HardwareX). It is also easier to troubleshoot problems, as most open-electronics applications are built on similar and wide-spread building blocks (i.e., Arduino platform) that share a common programming language, and large online communities exist that can be consulted to help solve specific problems (e.g., stackoverflow.com and raspberrypi.org/forums, with >300k members).

#### Transferable skills

Besides providing practical benefits, learning to work with open electronics and creating custom devices and applications also provides researchers with transferable skills, including knowledge of programming and electronics, technical problem solving, and creative thinking, which is paramount to scientific progress. This new skill set may spark new and cross-disciplinary ideas for research. It can further generate engineering- or programming-related skills, which improve researchers’ employability outside academia or lead to unexpected business opportunities (e.g., the start-up ElectricBlue). It also enables researchers and institutional staff to become instrument generalists, who, once they obtain open-electronics skills, can operate, service, or modify different types of open-electronics setups with no or less training than for specialized proprietary instruments. Technicians and staff can then more flexibly work within and across departments and become more valuable for institutions.

### Benefits to departments and institutions

#### Access to customizable high-end applications

Access to cutting edge scientific instrumentation is key to the success of many research institutions. However, expenses to purchase, maintain, or modify high-end instruments are often prohibitive. Open electronics have advanced to cutting-edge scientific instrumentation and provide the added benefit of being lower cost and highly customizable. There are numerous examples of open-electronics instruments with uncompromising quality being used for high-end scientific research, such as automated microbiological incubators to study adaptive evolution (Wong et al. 2018), high-throughput tracking and optogenetic stimulations to study fly's sensorimotor behavior (Tadres and Louis 2020), and microfluidic single cell sequencing preparation (Stephenson et al. 2018). Academic institutions can benefit from open-electronics solutions as a leaner way to perform workflows in-house through a modular, gradual investment, overcoming the need for researchers to depend on large grants or follow-up funding for instrumental maintenance and upgrades. Institutions can foster uptake by providing dedicated open-electronics workspaces, where researchers can implement ideas, and build institutional networks to share knowledge, ideas, and instruments across departments and stimulate interdisciplinary innovation.

#### Cost reductions and improved sustainability

When encouraged as an institutional-wide policy, the cost effectiveness of open electronics can be extended for the lifetime of the equipment. By supporting researchers to communicate and document about their open-electronics instruments and providing common guidelines, institutions can enhance the knowledge pool among staff and thereby help generate and support a “user-maker” community. This in turn not only facilitates the assembly and use of open-electronics instruments, but will help ensure that most maintenance, repairs, and upgrades can be performed in-house quickly, by the users themselves, with minimal fabrication expenses beyond parts. Such an approach provides the opportunity to practice a more sustainable small-scale circular economy concept at institutions (Prendeville et al. 2016) because of the ability to replace or update components of the hardware as opposed to disposing of entire units of outdated proprietary instruments. Such control over sustaining critical scientific equipment is crucial for all research institutions, but especially so for institutes with scarce funding or in countries where local technical support from specialized vendors lacks or is prohibitively expensive.

While small custom setups are most common among open-electronics projects, they are by no means limited to these. For example, in order to grow and maintain their large infrastructure sustainably, the European Center for Nuclear Research (CERN) built the electronic components of their particle accelerator with open-source hardware (van der Bij et al. 2012). Following this uncommon path, they have been simultaneously innovating in electronics CAD software KiCAD and the CERN Open Hardware License (Svorc and Katz 2019) as well as including openness as a requirement of their manufacturing contracts, thereby avoiding vendor lock-in. One resource example which was developed in this context but is now used across academia and industry, is the White Rabbit, the current gold standard to achieve ultra-fast data transfer synchronization in Ethernet networks (Moreira et al. 2009).

#### Improved collaboration and visibility

Open electronics can facilitate collaborations within and across institutions through the collective development and implementation of open-electronics solutions for frontier research applications. Institutions can foster this by intra- and inter-institutional think-tanks, workspaces, and shared educational programs, technical support as well as attributing dedicated time to researchers. The diverse application range of open electronics enhances cross-disciplinary collaboration potential but requires shared benefits for all parties. If successful, collaborations with, for example, engineers may enable highly complex and broadly useful open-electronics applications. Resulting publications, where useful tools are published in addition to research data, will likely have higher impact and citation rates similar to open data (Colavizza et al. 2020). A clear commitment to technologies that democratize science will also help institutions to enhance collaborations between industrialized and emerging nations and attract researchers that can easily cross-transfer open-electronics technologies. Potentially, this will not only improve institutes’ international visibility and reputation but may also help in acquiring public funding.

### Benefits to the scientific community and funders

#### Improved transparency and reproducibility

Transparency and reproducibility are hallmarks of the scientific method, but high costs and lack of documentation of procedures and tools in published methods commonly prevent effective replication (Baker 2016). Open electronics offer an opportunity to counter this issue. Published applications based on open electronics become technically and financially easier to reproduce through decreased reliance on proprietary solutions. At the same time, as more new developments about open electronics are published, the more it will become accepted and established to transparently communicate detailed blueprints of the solutions employed.

#### Decoupling of research capability from funding

The user-centered and iterative nature of open electronics better facilitates and enables specialized research than most proprietary solutions. For example, the use of electronics in biological research in harsh ecosystems, such as wave-swept rocky shores or remote deserts, is difficult, and equipment that is not specifically designed for it may easily become damaged or lost. In this context, researchers either secure more funds to cover the losses of expensive material or down-scale the research line. Alternatively, open electronics can be efficiently harnessed to develop cheaper and fully fit-for-purpose equipment (Gandra et al. 2015), while minimizing the cost incurred when losses occur. These and equivalent solutions alleviate the entry cost of many research topics and contribute to a greater decoupling of research capability from funding, ultimately facilitating the exploration of novel research lines and supporting investigations of early career researchers and scientists worldwide, which have reduced access to infrastructure and funding.

#### High innovation and collaboration potential

It is a common prejudice that open-source development conflicts with commercialization and industry collaboration. Just like successful open-source software companies, open electronics are an excellent basis for commercial knowledge transfer. Well-designed scientific instruments mutually involve developers (typically engineering-oriented teams but increasingly also open electronics “makers”) and end-users (typically non-engineering-oriented researchers) during the innovation and development process. However, often, such user-centered design is not achieved due to the lack or ineffective communication between both groups. Open electronics can overcome this by enabling end-users to become innovators and raising researchers’ basic electronics and programming skills to ease communication and collaboration between developers and scientific users.

Such additional technical skills by end-users ensure a better grasp of current technological boundaries, permitting the establishment of goals that are simultaneously realistic and ambitious. This can even lead to new commercial products. For example, some now-commercial hardware options in the field of ecology came from in-house custom laboratory solutions (e.g., Audiomoth [Open Acoustic Devices, Southampton, UK], ElectricBlue [ElectricBlue, Porto, Portugal]). At the same time, this user-centered and iterative development approach speeds up development cycles, which often results in fully functional solutions, and in many cases, it further enhanced by free user contributions. Those and further advantages (e.g., fast-adaptation, easy user engagement, and advertisement) can outweigh the disadvantages of such open-source business models (e.g., reduced profit timeframe after innovation cycles are stopped, less acceptance of excessive price margins) and provide rewarding opportunities for commercial developers and scientific users alike (Pearce 2017).

#### Bidirectional knowledge transfer between public and science

While an increasing number of scientists have been inspired to integrate many of the openly shared electronics solutions (e.g., home applications such as surveillance and home automation) into scientific experiments (Jolles 2021a), it also offers great opportunity to facilitate bidirectional collaborations with the public and science. Funders and society increasingly expect scientists to engage more actively with the public to improve the uptake and application of scientific knowledge (Hunter 2016). At the same time, the public increasingly demands to actively engage in the scientific process, to an extent that citizens partner or even co-author with professional scientists (Breen et al. 2015; Mazumdar et al. 2017). However, access to scientific instruments has partly hampered such engagements and bottom-up initiatives by non-scientists to develop scientific questions themselves (Mazumdar et al. 2017; Ostermann‐Miyashita et al. 2021). Open electronics can overcome this barrier by providing cost-effective and interactive tools that can be easily rebuilt by non-experts while having the capacity to generate high-quality scientific data (Weeser et al. 2018). The user-centered, iterative, and modular design that is inherent to open-electronics solutions further enhances a smooth exchange of knowledge and technical solutions between professional and community scientists. Established design-sharing platforms such as Thingiverse enable direct feedback from public communities and potentially accelerate the evolution of scientific open-electronics equipment. Thus, open electronics are well suited to make science broadly reproducible and more accessible for new collaboration opportunities.

## Barriers and trade-offs

To reap the remarkable potential of open electronics for biological research and science in general, significant educational, collaborative, and technical barriers need to be overcome. For example, while the Raspberry Pi has large application potential, most researchers still lack basic awareness of such devices and their capacities and hence their uptake remains limited (Jolles 2021a). One major reason is the lack or insufficient documentation of open-electronics setups in scientific publications, which confines its visibility and the formation of any substantial academic “Maker” community (Glenn and Alfredo 2010; Harnett 2011). Instead, many open-electronics techniques are spread among collaborators in an informal fashion with limited reach. However, initiatives exist that aim to increase the visibility of open hardware solutions, such as the Open Neuroscience network (Maia Chagas 2021), the Open Source Ecology, Wildlab.net, and Conservation X Labs (see Table 2), or new journals documenting open hardware designs in a systematic fashion, like the Journal of Open Hardware (Murillo and Wenzel 2017) and HardwareX.

Further, open electronics are poorly visible in disciplines where they do not define the research goal. For example, a Web of Science search showed that engineers dominate the use of open-electronics boards listed in Fig. 3 (36.1%), followed by Computer Science (26.4%, Fig. 1B, Supplementary Information File S1). However, a more detailed full-text analysis showed that although biological sciences dominate the use of these boards within PLOS journals (34%, *n* = 86), before engineering (11%, *n* = 27), only 5% of these articles reported their use in the abstract (for detailed analysis, see Supplementary Information File S2). In addition, authors do not always mention clearly if and how they applied open electronics in their research, making it difficult to identify its actual use across subject areas. With improved acknowledgment, researchers will recognize its value at a broader scale and potentially generate more associated research, innovation, and public interest.

Another barrier is the fragmentation of the existing open-electronics user community within institutions and across countries and subject domains, hindering the exchange and consolidation of knowledge (Fig. 1A and B). This is because within institutions and departments, there is often little support infrastructure for development, educational resources, and community-building, such as user-run “Maker” workshops (Maia Chagas 2018). Across countries, peer-to-peer knowledge exchange is largely limited to existing collaboration networks, often biased by geographical distance or socio-cultural cohesion (Hennemann et al. 2012), although open electronics’ scientific publication output, led by and linked between China, the United States, and India, indicates promising shifts to break those traditional patterns (Fig. 1A, Supplementary Information File S1).

Other important trade-offs of open electronics are time investment to learn and prototype, difficulties to access or sustain expertize and knowledge, or the sustained ability to operate and maintain equipment. These need to be weighed against the benefits and trade-offs posed by proprietary alternatives, researchers’ needs to access customized solutions, and their individual time or financial limitations. For example, if there are affordable proprietary solutions that fulfill the research need (e.g., temperature measurement), then open electronics may not provide substantial benefits. Also, more complex setups may require significant time investments for optimization, repair, and maintenance, particularly during early development stages, which may outweigh their initial lower costs compared to well-tested and established commercial alternatives. However, once the need for customization increases (e.g., additional sensors with cloud data transmission), the access to suitable commercial alternatives declines and their price tends to increase. Much research requires highly customized solutions without providing the quantitative scale for commercial vendors to manufacture low-priced options. Thus, researchers can choose to purchase costly niche products, contract companies to develop and repair customized applications at significant costs, or access other available resources such as an institute's workshop and engineering staff. While the latter two can be time-consuming too, many researchers lack access to the necessary funding, local specialized companies, or infrastructure support. For them, open electronics may be the only pathway to execute the intended research with time investment being a relatively minor trade-off.

Local peer-knowledge can be key to shorten time investments for learning and development of open-electronics setups, particularly when complexity increases. However, skilled users within departments or institutions are still rare and collaborations with engineering scientists unlikely due to the lack of shared goals or benefits. Also, locally established knowledge may not sustain if scientists leave institutions. In contrast, companies normally provide good product documentation and customer support, yet more complex instrumentations require intense training at additional costs, and product support may cease for old products or when companies cease to exist. To improve the access and sustainability of local knowledge, good documentation, the establishment of local open-electronics user networks, and a structured knowledge transfer will be key. This will also support self-repair and maintenance of open-electronics equipment by users not involved in their development. In comparison, repair of proprietary products may become expensive once warranty expires and self-repair impossible due to sealed instruments, inaccessible hardware documentation, or surface mount electronics. Finally, while broad experience with and detailed knowledge of coding is still a common perceived barrier for people to get started with open electronics, for most projects, this is actually not required. Furthermore, researchers can rely on easy-to-use libraries to work with sensors and actuators and numerous online resources that provide tutorials with ready-to-paste code (Table 2), thereby significantly lowering the skill requirements for non-programmers. The discussed trade-offs may weigh differently depending on the individual knowledge access and research support and may decrease with time as skills and collaborations increase.

We believe that over time many of the discussed barriers and trade-offs can be offset, beginning by increasing the visibility of the tools themselves and a cultivation of an institutional and global collaborative community around their use, to build confidence, electronics literacy, and local knowledge pools. Global networks such as the Gathering for Open Science Hardware (Murillo et al. 2018) and Open-Source Ecology (see Table 2), and an increasing number of scientific societies hosting dedicated symposia (e.g., the Annual Meeting of the Society for Experimental Biology or the Society for Integrative and Comparative Biology), are an excellent start to spread awareness and share innovative open-electronics solutions across disciplines and budgets.

## Beginners toolbox

### Starting simple

**Figure 2.**
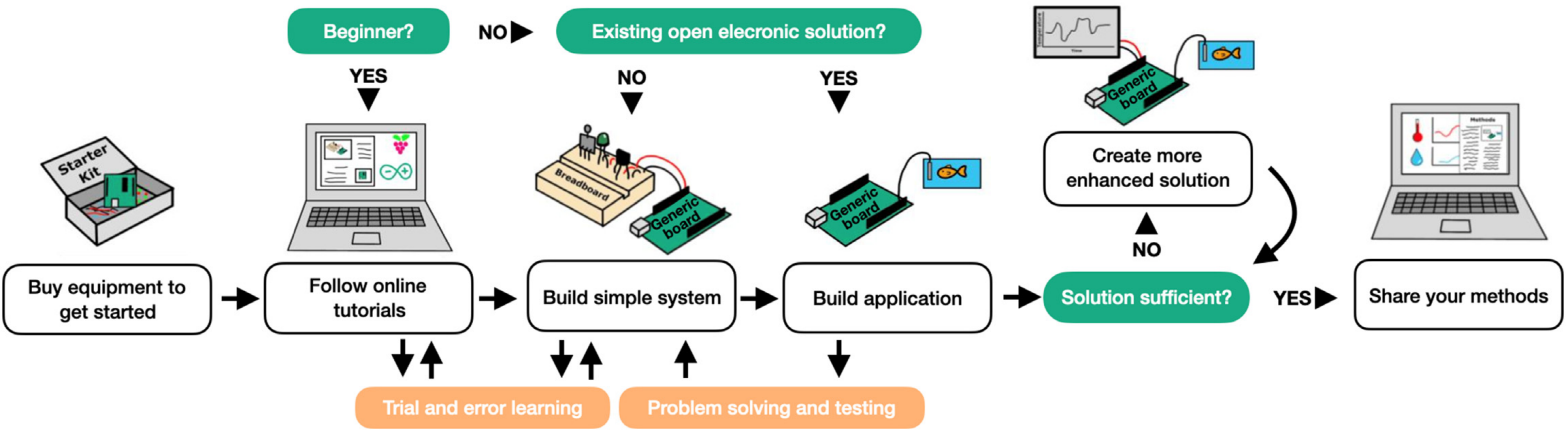
Diagrammatic representation of the potential steps for incorporating open electronics into one's research. It is best to begin with a starter kit to explore its potential. Tutorials are useful for building initial skills, such as to set up a sensor to measure the temperature of an aquarium. Delving further into the many (online) resources available, basic systems can be expanded to perform more advanced tasks, such as plotting the temperature data in real time on a simple website and sending warning emails whenever values cross thresholds. Such a system can then be easily and affordably replicated and shared with the broader community.

**Figure 3.**
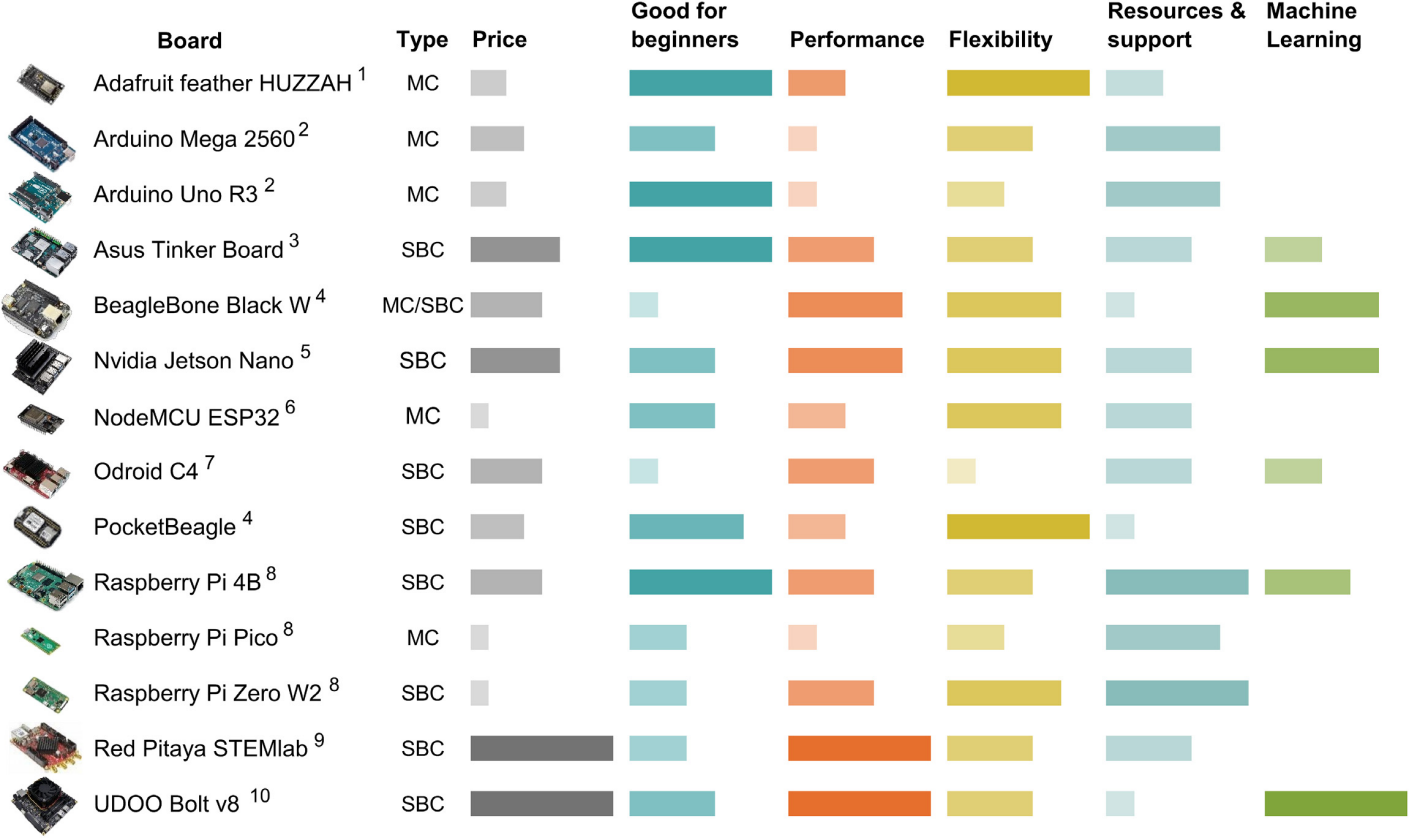
Overview of some of the key micro-controllers (MC) and single-board computers (SBC) on the market, designed to enable the creation of open-electronics applications by non-experts. Models are rated for their price, skill level, performance, flexibility, resources, and support available, and possibility to run machine learning applications, based on publicly available model specifications (dimensions, RAM, CPU, number of cores, GPU, power consumption, number of interfaces, wired and wireless connectivity, video recording capability, storage), popularity, and authors’ experiences. Manufacturers: ^1^Adafruit Industries, New York, USA; ^2^Arduino, New York, USA; ^3^ASUSTek Computer Inc., Taipei, Taiwan; ^4^Texas Instruments Incorporated, Dallas, USA; ^5^Nvidia Corporation, Santa Clara, USA; ^6^Espressif Systems, Shanghai, China; ^7^Hardkernel Co., Ltd., GyeongGi, South Korea; ^8^Raspberry Pi Foundation, Cambridge, UK; ^9^Red Pitaya, Solkan, Slovenia; ^10^Udoo, Arezzo, Italy.

Once the decision has been made to use open electronics, the best way to begin is to start creating simple set-ups, to gain experience in how open electronics work, and to inspire first custom applications (Fig. 2). Hobby electronics starter kits (e.g., www.sparkfun.com, www.adafruit.com) provide all essential electronic components, such as breadboard, cables, resistors, or LEDs, and introduce researchers to the large range of sensors and actuators useful in biological research (Table 1). These kits come with micro-controllers and single-board computers, for which a large range of options exist (see Fig. 3 for an overview of devices). While the micro-controllers are mostly constrained to one programming language (e.g., C/C++, micropython), single-board computers offer more programming choices (e.g., Python, C++, JavaScript etc.). For beginners especially, Arduino and Raspberry Pi are recommended as they have the most documentation and support available (see Table 2). One can then gradually progress to other more specialized applications and boards (Fig. 3), using the many additional introductory learning resources (Table 2).

### Implement and iterate to advanced applications

Once researchers obtained their first electronic and programming skills, open electronics can be employed in simple biological experiments. This can include field logging of temperature or humidity (Baker 2014), recording images or videos in experiments (Jolles 2020), or detecting wildlife using weatherproof camera traps (Droissart et al. 2021). At this level already, reproducibility and scalability are important to consider, so that initial designs can be expanded to increase complexity, throughput, and replication by other researchers. Simple designs help to easily replicate prototypes, reduce time for troubleshooting during operation, and lower reproduction costs. For example, this can lead to a gradual expansion, first using one camera system to phenotype plants (Tovar et al. 2018) up to large-scale and simultaneous high-throughput phenotyping of 1800 plants using 180 Raspberry Pis and cameras (Tausen et al. 2020). Such a stepwise expansion is advisable and may require some iterations of replacement or optimization of electronic components and programming code, for which fellow users may provide support in the numerous online forums (Table 2). Apart from scale, applications may also grow in complexity, as most open-electronics boards allow the connection of multiple and different types of sensors, cameras, and actuators, either directly or via easy-to-connect and easy-to-read breakout boards. For example, this enables researchers to measure multiple variables simultaneously in single setups such as moisture, temperature, or different types of gases in soil (Bitella et al. 2014; Dorji et al. 2017); remotely analyze water bodies and landscapes using multispectral sensors carried by drones (Bokolonga et al. 2016); or monitor the bio-acoustic environment in tropical or temperate habitats (Whytock et al. 2016). A large variety of C or Python libraries enable the increase of application complexity beyond hardware without professional programming skills to, for example, send automatic email notifications, share and visualize data life, program custom GUIs to control electronics and devices, or analyze “data on board,” such as the simultaneous behavioral monitoring of up to 1400 flies, using machine learning algorithms (Geissmann et al. 2017) or constructing detailed 3D models of arthropods (Plum and Labonte 2021). Such complex applications may seem daunting for beginners, but an increasing number of traditional as well as dedicated open hardware scientific journals (e.g., Journal of Open Hardware or HardwareX) offer detailed construction and coding instructions to ease reproduction.

### Acknowledge and share open electronics

**Figure 4.**
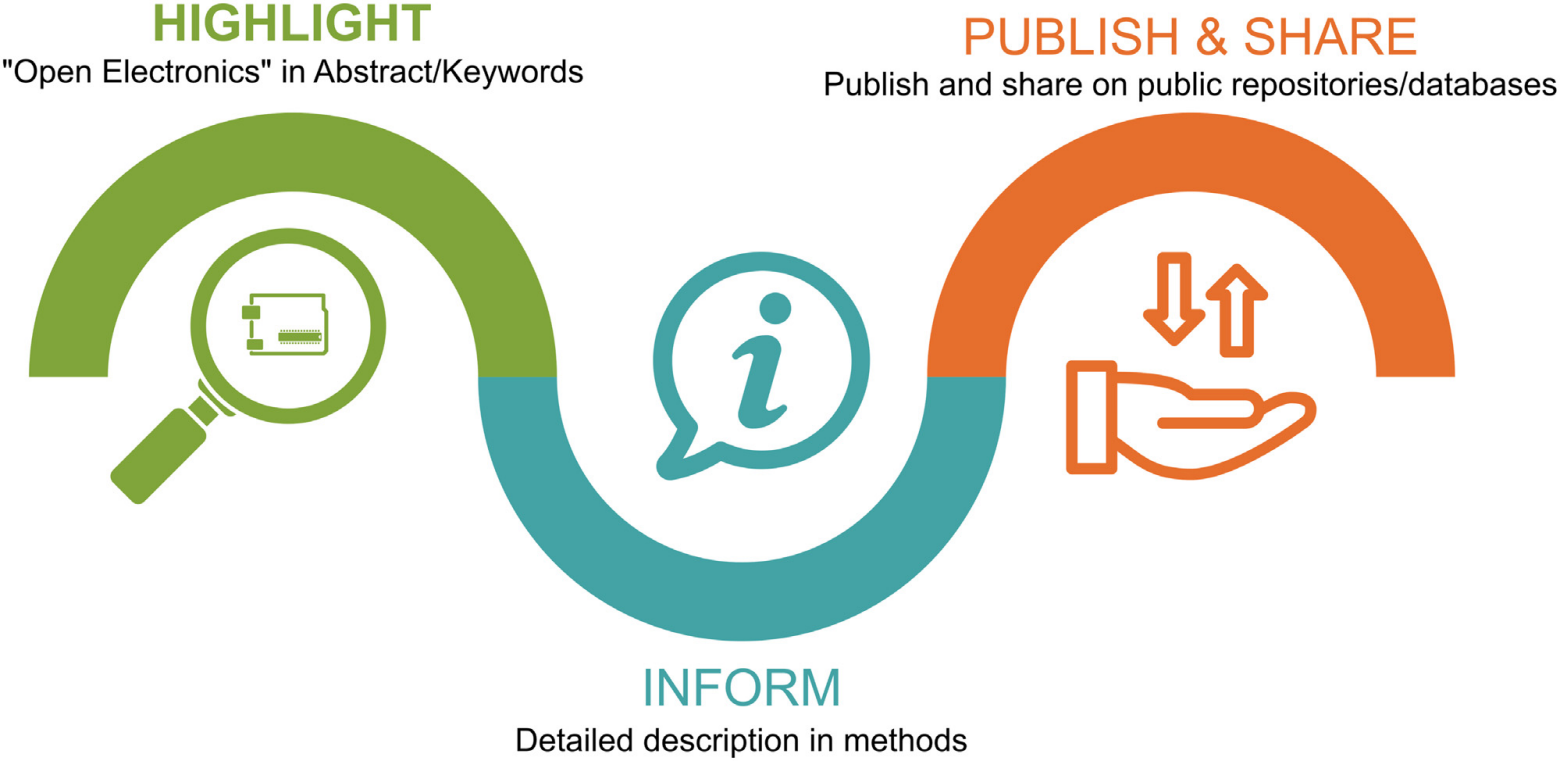
Concept diagram illustrating a three-step standard practice to best acknowledge the use of open electronics in science. Vector graphic courtesy of Arto Lereh Saraga, Jaohuarye, Uizin, and Adrien Coquet from NounProject.com.

For beginners and advanced users alike, good documentation is key to enable others to reproduce setups and further improve and adapt open-electronics designs. Therefore, it is essential that researchers properly acknowledge the use of open electronics and share their designs, methodologies, and knowledge with the broader community. This can be best achieved by following a three-step standard practice to acknowledge open-electronics solutions in research publications (Fig. 4).

1. The use of “open electronics” or “open hardware” should be clearly highlighted and mentioned using standard terms in the abstract and keywords. This enables users to easily locate relevant publications in public databases and metadata searches. Due to the large diversity of boards and new developments, simply using, for example, micro-controller names will not be comprehensive enough to locate all relevant sources. The introduction too should contain a short explanation of the open-electronics application.
2. The methods section should contain detailed information describing the open-electronics application. Those details should enable to fully replicate the setup and therefore include a list of all components with their model, supplier, and price; written or illustrated fabrication instructions; and photos, illustrations, 3D files, or videos (e.g., Eggert et al. 2020), and computer aided design (CAD) files where applicable. If methods are not the focus of the study and space is limited, then this section should include minimum a list of all components used with the exact model and supplier and a reference to the detailed constructions blueprint in the supplements or external sources.
3. In addition to providing those details in the methods section or supplements, one should publish and share projects on online platforms such as GitHub using Markdown files, on institutional or public Wikis, or even create a dedicated website using free services such as GitHub pages or WordPress. This has the benefit of receiving direct feedback from other users to improve and further develop the application. To ensure reproducibility of the published version, the exact version of this detailed documentation should be archived on a scientific webpage capable of generating a digital object identifier (DOI), such as Zenodo.org and this link should be shared in the publication itself. Depending on its novelty, one may also decide to write up a methods paper about the specific device and its applications, such as in the mentioned open hardware journals. This has the added benefit of creating a citable reference, which can improve publication output and researcher's visibility outside their own discipline, as well as serve as a proof of prior art of intellectual property.

## Outlook

The potential of open-electronics applications is endless and can greatly benefit individual researchers, institutions, and the scientific community in a broad variety of ways. With the increasing capabilities of electronic components and sensors, and computers becoming more powerful at decreasing size and cost, open electronics are likely to become progressively used and integrated in our day-to-day life, and over time become a standard component of the scientific toolbox. This, in turn, will result in new and cutting-edge technologies to be implemented at a broader scale, and help tech-innovation to expand to other non-engineering disciplines.

An important step to increase the uptake of open electronics is improved support by funding organizations, such as to grant researchers dedicated time to develop, build, publish open-electronics applications, and request open hardware alternatives in compulsory instrument bids. Furthermore, institutions can foster local “ScienceMaker” communities, by providing institutional “MakerHubs” or workshops, where researchers can prototype and exchange knowledge and ideas with others, as well as include electronics and programming training to the institutional career development portfolio. And scientific communities can start or join open hardware initiatives, for example, Global Open Science Hardware community (Murillo et al. 2018); organize dedicated conferences, sessions, or workshops to form networks; create standards; and foster open electronics across disciplines (Bonvoisin et al. 2020).

In this paper, we presented the multi-facetted benefits open electronics may offer to researchers, institutions, and the scientific community, and highlighted their utility and potential for biological research and science as a whole, while also noting important barriers and trade-offs, and avenues to overcome those, including a beginner's guide. We hope our review will help foster a broader awareness and uptake of open electronics across the biological sciences, and from research to education and outreach, and thereby to ultimately help increase the innovation, reproducibility, and democratization of science.

## Supplementary Material

icac043_Supplemental_Files

## Figures and Tables

**Figure:**
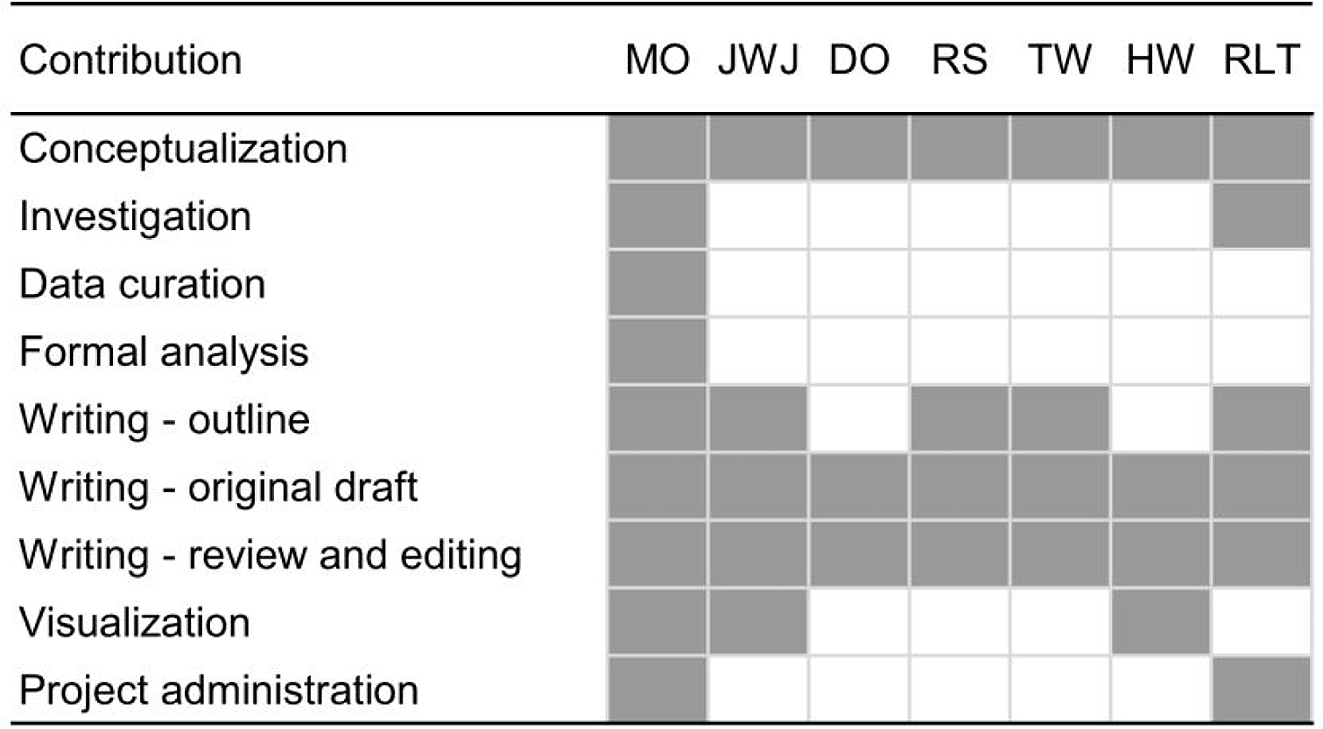


## Notes

From the symposium “Open source solutions in experimental design’’ presented at the annual meeting of the Society for Integrative and Comparative Biology virtual annual meeting, January 3–February 28, 2022.
