# Supplementary material for: Open Hardware in Science: The Benefits of Open Electronics
Source: Integr Comp Biol. 2022 May 20;62(4):1061–75. doi: 10.1093/icb/icac043 (PMC9617215; doi:10.1093/icb/icac043)

Country collaboration network for open electronics
Web of Science search results

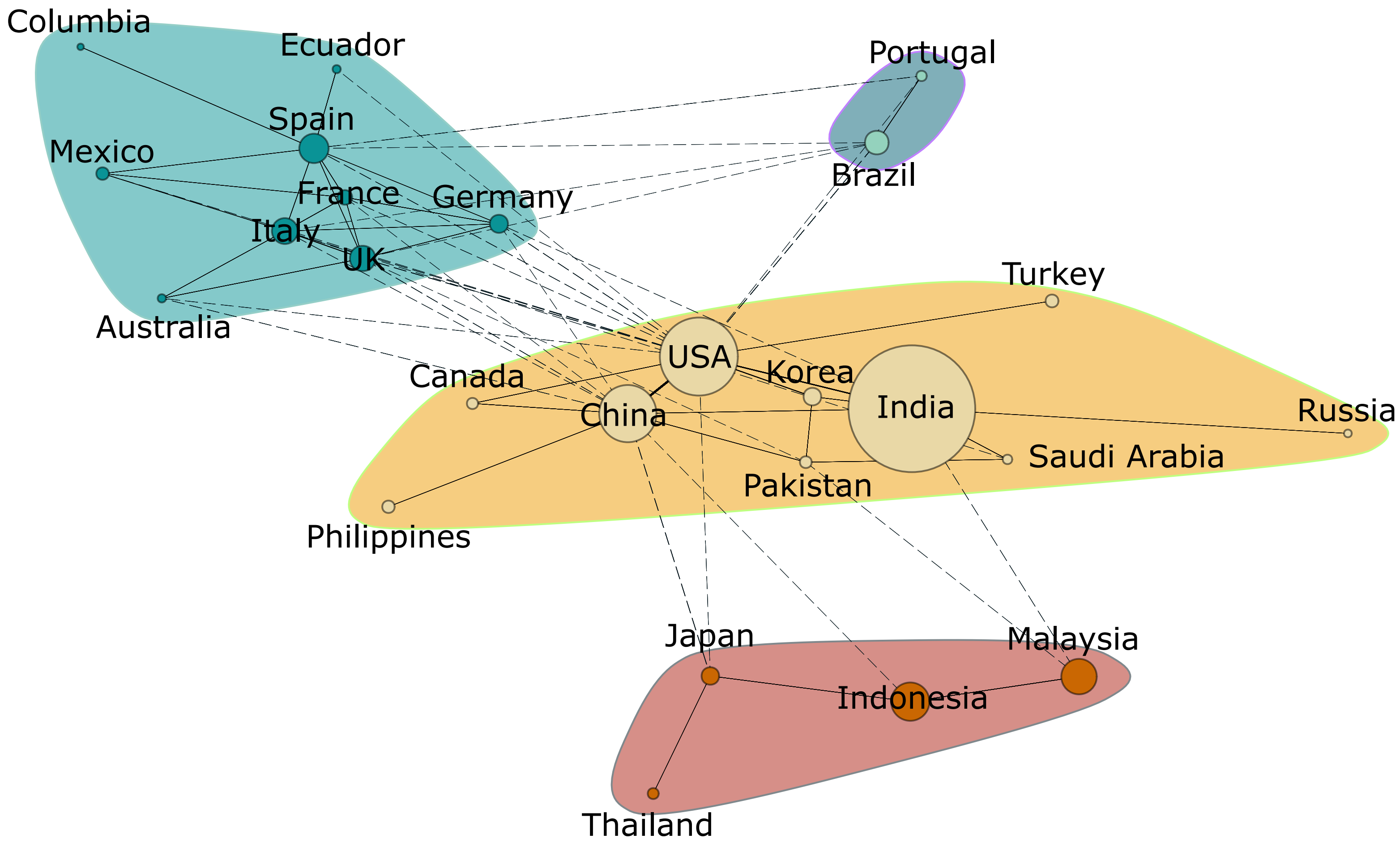

Supplement: icac043_Supplemental_Files [file icac043_supplemental_files.zip › icb-2022-0062-File009.zip!/File_S1_SuppInfo/CountryCollaborationNetwork.pdf]
