# Supplementary material for: Open Hardware in Science: The Benefits of Open Electronics
Source: Integr Comp Biol. 2022 May 20;62(4):1061–75. doi: 10.1093/icb/icac043 (PMC9617215; doi:10.1093/icb/icac043)
Supplement: icac043_Supplemental_Files [file icac043_supplemental_files.zip › icb-2022-0062-File009.zip!/File_S1_SuppInfo/Subjects_BarChart.pdf]

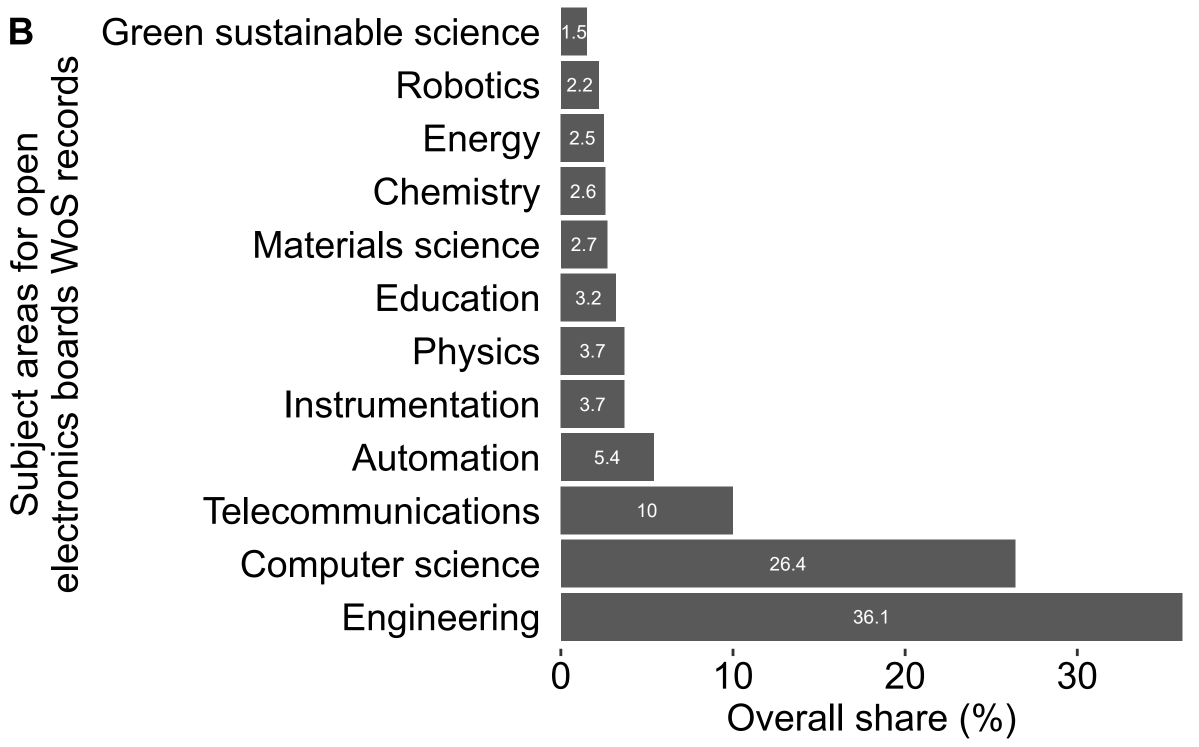
B
Subject areas for open electronics boards WoS records
Green sustainable science
1.5
Robotics
2.2
Energy
2.5
Chemistry
2.6
Materials science
2.7
Education
3.2
Physics
3.7
Instrumentation
3.7
Automation
5.4
Telecommunications
10
Computer science
26.4
Engineering
36.1
0
10
20
30
Overall share (%)
