# Supplementary material for: Open Hardware in Science: The Benefits of Open Electronics
Source: Integr Comp Biol. 2022 May 20;62(4):1061–75. doi: 10.1093/icb/icac043 (PMC9617215; doi:10.1093/icb/icac043)
Supplement: icac043_Supplemental_Files [file icac043_supplemental_files.zip › icb-2022-0062-File009.zip!/File_S1_SuppInfo/Subjects_CumulativeGrowthByYear.pdf]

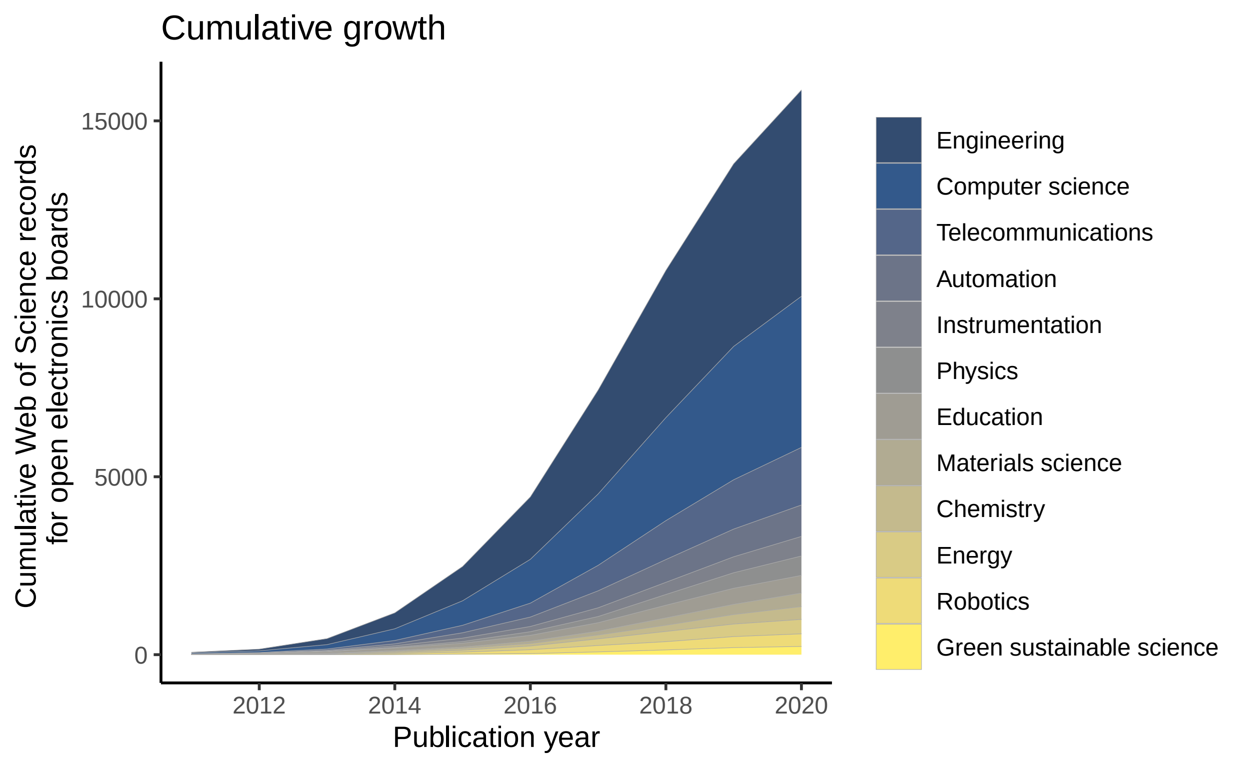

Cumulative growth
Cumulative Web of Science records for open electronics boards
15000
10000
5000
0
2012
2014
2016
2018
2020
Publication year
Engineering
Computer science
Telecommunications
Automation
Instrumentation
Physics
Education
Materials science
Chemistry
Energy
Robotics
Green sustainable science
