# Supplementary material for: Open Hardware in Science: The Benefits of Open Electronics
Source: Integr Comp Biol. 2022 May 20;62(4):1061–75. doi: 10.1093/icb/icac043 (PMC9617215; doi:10.1093/icb/icac043)
Supplement: icac043_Supplemental_Files [file icac043_supplemental_files.zip › icb-2022-0062-File009.zip!/File_S1_SuppInfo/Top_Countries_Cumulative.pdf]

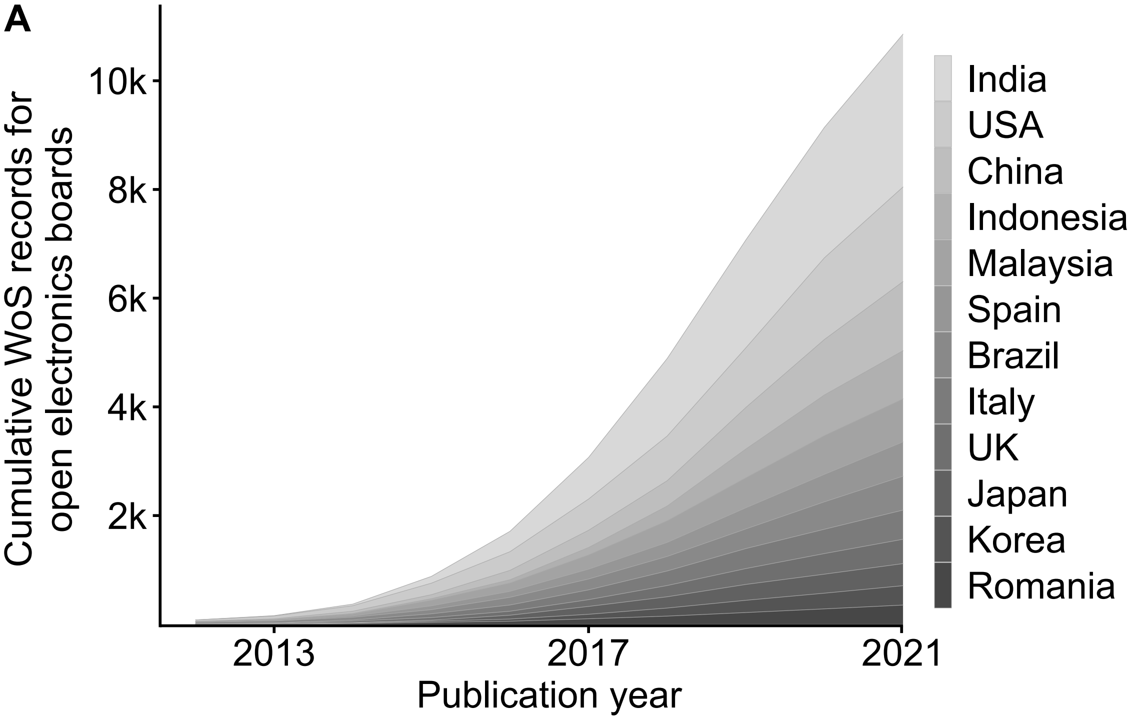
A
Cumulative WoS records for open electronics boards
10k
8k
6k
4k
2k
2013
2017
2021
Publication year
India
USA
China
Indonesia
Malaysia
Spain
Brazil
Italy
UK
Japan
Korea
Romania
