# Supplementary material for: Open Hardware in Science: The Benefits of Open Electronics
Source: Integr Comp Biol. 2022 May 20;62(4):1061–75. doi: 10.1093/icb/icac043 (PMC9617215; doi:10.1093/icb/icac043)
Supplement: icac043_Supplemental_Files [file icac043_supplemental_files.zip › icb-2022-0062-File009.zip!/File_S1_SuppInfo/WoS_LiteratureSearch.html]

Bibliographic analysis for open electronics Web of Science records


# Bibliographic analysis for open electronics Web of Science records

#### Michael Oellermann

#### 21 July, 2021

## Search query informations

### Search term

TOPIC: (Arduino) OR TOPIC: (“Asus Tinker Board”) OR TOPIC: (“BeagleBone Black”) OR TOPIC: (“Adafruit feather HUZZAH”) OR TOPIC: (“Nvidia Jetson Nano”) OR TOPIC: (“Odroid C4”) OR TOPIC: (PocketBeagle) OR TOPIC: (“Raspberry Pi”) OR TOPIC: (“Red Pitaya”) OR TOPIC: (“UDOO Bolt”) Timespan: 2007-2021. Indexes: SCI-EXPANDED, SSCI, CPCI-S, CPCI-SSH. Article type: articles, proceedings papers and reviews

Search performed following instructions at: https://www.bibliometrix.org/vignettes/Introduction\_to\_bibliometrix.html Web of Science link accessed: https://www.webofknowledge.com

# 1. Load libraries

# 2. Load data

```
# # File path
wd <- "C:/Users/oelle/Documents/Meine Dokumente/Research/Publications and Reports/2021/Open Electronics/LiteratureSearch/OE_LiteratureSearch/SearchAllTerms/"

#Load raw files and merge to single data frame
# Locate and list files
# wos_files <- list.files(path = wd, pattern = "*.txt",full.names = TRUE)
# 
# # Load files into one bibliography
# M <- convert2df(file = wos_files, dbsource = "isi", format = "plaintext")

# # Save file
#   # As CSV
#     # write.csv(M, "OE_WoS_LitSearch.csv")
#   # As R data format
#     saveRDS(M, file = "OE_WoS_LitSearch.rds")
    
M <- readRDS(paste(wd, "OE_WoS_LitSearch.rds", sep = ""))
```

# 4. Descriptive analysis

```
# # Short data summary
#   results <- biblioAnalysis(M, sep = ";")
#  
# # Save results
#   saveRDS(results, file="OE_WoS_LitSearch_Results.rds")

# Load results
  results <- readRDS(paste(wd, file = "OE_WoS_LitSearch_Results.rds", sep = ""))

# Summarise data
  options(width=100)
  S <- summary(object = results, k = 10, pause = FALSE)
```

```
## 
## 
## MAIN INFORMATION ABOUT DATA
## 
##  Timespan                              2007 : 2021 
##  Sources (Journals, Books, etc)        4949 
##  Documents                             10360 
##  Average years from publication        3.5 
##  Average citations per documents       2.85 
##  Average citations per year per doc    0.6046 
##  References                            149853 
##  
## DOCUMENT TYPES                     
##  article                         2148 
##  article; data paper             1 
##  article; early access           68 
##  article; proceedings paper      66 
##  biographical-item               1 
##  book review                     1 
##  correction; early access        1 
##  editorial material              33 
##  meeting abstract                34 
##  news item                       4 
##  proceedings paper               7973 
##  reprint                         1 
##  retraction                      1 
##  review                          26 
##  review; early access            2 
##  
## DOCUMENT CONTENTS
##  Keywords Plus (ID)                    4129 
##  Author's Keywords (DE)                20889 
##  
## AUTHORS
##  Authors                               31327 
##  Author Appearances                    40286 
##  Authors of single-authored documents  401 
##  Authors of multi-authored documents   30926 
##  
## AUTHORS COLLABORATION
##  Single-authored documents             481 
##  Documents per Author                  0.331 
##  Authors per Document                  3.02 
##  Co-Authors per Documents              3.89 
##  Collaboration Index                   3.13 
##  
## 
## Annual Scientific Production
## 
##  Year    Articles
##     2007        2
##     2008        6
##     2009        8
##     2010       16
##     2011       41
##     2012       73
##     2013      201
##     2014      481
##     2015      844
##     2016     1254
##     2017     1782
##     2018     2031
##     2019     1901
##     2020     1281
##     2021      368
## 
## Annual Percentage Growth Rate 45.13518 
## 
## 
## Most Productive Authors
## 
##    Authors        Articles Authors        Articles Fractionalized
## 1    KUMAR A            44       KUMAR A                    11.20
## 2    KUMAR S            25       BOGDAN M                    9.00
## 3    BAROLLI L          24       URIEN P                     8.00
## 4    ODA T              22       KUMAR S                     7.34
## 5    LEE S              20       LEE S                       6.35
## 6    KHAN S             18       GUO G                       5.92
## 7    SHARMA A           18       SUNG WT                     5.67
## 8    WANG Y             18       SHARMA A                    5.48
## 9    CASTRO M           17       CRAFT B                     5.00
## 10   DELA CRUZ JC       17       NUSSEY J                    5.00
## 
## 
## Top manuscripts per citations
## 
##                                                                                                                       Paper         
## 1  LU HM, 2018, IEEE INTERNET THINGS                                                                                                
## 2  BUECHLEY L, 2008, CHI 2008: 26TH ANNUAL CHI CONFERENCE ON HUMAN FACTORS IN COMPUTING SYSTEMS VOLS 1 AND 2, CONFERENCE PROCEEDINGS
## 3  OUADDAH A, 2016, SECUR COMMUN NETW                                                                                               
## 4  D'AUSILIO A, 2012, BEHAV RES METHODS                                                                                             
## 5  COSTANTINE J, 2015, P IEEE                                                                                                       
## 6  FERDOUSH S, 2014, PROCEDIA COMPUT SCI                                                                                            
## 7  GUO G, 2012, IEEE T VEH TECHNOL                                                                                                  
## 8  PIEDRAHITA R, 2014, ATMOS MEAS TECH                                                                                              
## 9  LI JP, 2014, ACS NANO                                                                                                            
## 10 ZHANG YY, 2019, IEEE INTERNET THINGS                                                                                             
##                            DOI  TC TCperYear   NTC
## 1  10.1109/JIOT.2017.2737479   220      55.0 93.07
## 2  NA                          196      14.0  3.73
## 3  10.1002/sec.1748            188      31.3 49.37
## 4  10.3758/s13428-011-0163-z   181      18.1 15.84
## 5  10.1109/JPROC.2015.2396000  172      24.6 38.00
## 6  10.1016/j.procs.2014.07.059 172      21.5 27.67
## 7  10.1109/TVT.2012.2203362    129      12.9 11.29
## 8  10.5194/amt-7-3325-2014     125      15.6 20.11
## 9  10.1021/nn506610p           115      14.4 18.50
## 10 10.1109/JIOT.2018.2847705   110      36.7 65.20
## 
## 
## Corresponding Author's Countries
## 
##           Country Articles   Freq  SCP MCP MCP_Ratio
## 1  INDIA              1973 0.1925 1900  73    0.0370
## 2  USA                 969 0.0945  862 107    0.1104
## 3  CHINA               732 0.0714  636  96    0.1311
## 4  INDONESIA           594 0.0580  557  37    0.0623
## 5  MALAYSIA            484 0.0472  434  50    0.1033
## 6  SPAIN               349 0.0340  288  61    0.1748
## 7  BRAZIL              330 0.0322  290  40    0.1212
## 8  ITALY               297 0.0290  246  51    0.1717
## 9  UNITED KINGDOM      259 0.0253  199  60    0.2317
## 10 ROMANIA             251 0.0245  232  19    0.0757
## 
## 
## SCP: Single Country Publications
## 
## MCP: Multiple Country Publications
## 
## 
## Total Citations per Country
## 
##       Country      Total Citations Average Article Citations
## 1  USA                        4594                     4.741
## 2  INDIA                      3614                     1.832
## 3  CHINA                      2672                     3.650
## 4  SPAIN                      1872                     5.364
## 5  ITALY                      1837                     6.185
## 6  UNITED KINGDOM             1417                     5.471
## 7  KOREA                      1154                     4.869
## 8  MALAYSIA                    968                     2.000
## 9  BRAZIL                      811                     2.458
## 10 CANADA                      605                     4.764
## 
## 
## Most Relevant Sources
## 
##                                                                                                                                             Sources       
## 1  SENSORS                                                                                                                                                
## 2  IEEE ACCESS                                                                                                                                            
## 3  ELECTRONICS                                                                                                                                            
## 4  IFAC PAPERSONLINE                                                                                                                                      
## 5  APPLIED SCIENCES-BASEL                                                                                                                                 
## 6  MATERIALS TODAY-PROCEEDINGS                                                                                                                            
## 7  IEEE SENSORS JOURNAL                                                                                                                                   
## 8  IEEE LATIN AMERICA TRANSACTIONS                                                                                                                        
## 9  ADVANCED SCIENCE LETTERS                                                                                                                               
## 10 2018 IEEE 10TH INTERNATIONAL CONFERENCE ON HUMANOID NANOTECHNOLOGY INFORMATION TECHNOLOGY COMMUNICATION AND CONTROL ENVIRONMENT AND MANAGEMENT (HNICEM)
##    Articles
## 1       208
## 2        86
## 3        75
## 4        56
## 5        54
## 6        47
## 7        42
## 8        37
## 9        34
## 10       30
## 
## 
## Most Relevant Keywords
## 
##    Author Keywords (DE)      Articles Keywords-Plus (ID)     Articles
## 1   ARDUINO                      2051            SYSTEM           269
## 2   RASPBERRY PI                 1446            DESIGN           194
## 3   INTERNET OF THINGS            567            INTERNET         193
## 4   IOT                           525            THINGS           112
## 5   ARDUINO UNO                   272            PERFORMANCE       93
## 6   SENSORS                       265            MODEL             72
## 7   MICROCONTROLLER               248            SYSTEMS           70
## 8   IMAGE PROCESSING              172            SENSOR            67
## 9   INTERNET OF THINGS (IOT)      166            ARDUINO           60
## 10  ANDROID                       160            MANAGEMENT        52
```

# 5. Plot results

```
# plot(x = results, k = 10, pause = FALSE)
```

# 6. Country statistics

```
# Extract metatags and attach to bibliometric data frame
  M <- metaTagExtraction(M, Field = "AU_CO", sep = ";")

# Subset to articles and proceedings papers only
  M <- M[grep(pattern = "ARTICLE|PROCEEDINGS PAPER", M$DT),]
  
# # Subset to proceedings papers only
#   M <- M[grep(pattern = "PROCEEDINGS PAPER", M$DT),]
 
# Country growth
  #cdf = TRUE means cumulative growth
  top_countries <- KeywordGrowth(M, Tag = "AU_CO", sep = ";", top = 12, cdf = TRUE)

# Convert country labels to sentence case
  names(top_countries)[c(2, 4:ncol(top_countries))] <- to_any_case(names(top_countries)[c(2, 4:ncol(top_countries))], case = "sentence")
  
# Correct upper case for UK
  names(top_countries)[grep("kingdom", names(top_countries))] <- "UK"
  names(top_countries)[grep("Usa", names(top_countries))] <- "USA"

# Plot area chart by country
  top_countries_long <- melt(top_countries, id='Year')
  # Add numerical value country column for color scale
    # Create color range
      colfunc <- colorRampPalette(c("#cfcfcf", "#1a1a1a"))   ##2C4D78", "#FBB957"
      country_colors <- colfunc(length(levels(top_countries_long$variable)))

  ggplot(subset(top_countries_long, Year > 2010 & Year < 2021), aes(Year,value, 
                                 group=variable, 
                                 fill=variable))+
    geom_area(alpha=0.8, color='darkgrey', size = 0.1) +
    labs(x="Publication year", y='Cumulative WoS records for \n open electronics boards', fill = "")+
    scale_fill_manual(values=country_colors)+
    theme_classic()+
    theme(text = element_text(size = 14), axis.text = element_text(size = 14, color = "black"), legend.text = element_text(size = 14))+
    scale_x_continuous(breaks=seq(2008, 2020, 4), labels = seq(2009, 2021, 4))+
    scale_y_continuous(breaks=seq(2000, 10000, 2000), labels = paste(seq(2, 10, 2), "k", sep = ""))
```

```
  ggsave(paste(wd, "Top_Countries_Cumulative.pdf", sep = ""), width = 6.5, height = 4)
  ggsave(paste(wd, "Top_Countries_Cumulative.svg", sep = ""), width = 6.5, height = 4)
  
  # For proceedings only
  # ggsave(paste(wd, "Top_Countries_Cumulative_Proceedings.pdf", sep = ""), width = 6.5, height = 4)
  # ggsave(paste(wd, "Top_Countries_Cumulative_Proceedings.svg", sep = ""), width = 6.5, height = 4)
```

# 7. Subject analysis

```
# Split strings by semicolon into separate columns
  subject <- data.frame(M$WC)
  subject <- separate(data = subject, col = M.WC, into = c("a", "b", "c", "d", "e", "f", "g"), sep = ";", fill = "right", extra = "warn")

# Group subject by major science domains
  pattern_x <- c("COMPUTER SCIENCE",
                 "ENGINEERING",
                 "AUTOMATION",
                 "ENERGY",
                 "CHEMISTRY",
                 "EDUCATION",
                 "GREEN & SUSTAINABLE SCIENCE",
                 "TELECOMMUNICATIONS",
                 "MATERIALS SCIENCE",
                 "INSTRUMENT",
                 "OPTIC",
                 "BIOCHEMIC",
                 "AGRICULTUR",
                 "ARCHITECTUR",
                 "PHYSIC",
                 "ENVIRONMENTAL",
                 "ECOLOGY",
                 "BIOLOGY",
                 "IMAGING SCIENCE")
  replacement_x <- c("COMPUTER SCIENCE",
                     "ENGINEERING",
                     "AUTOMATION",
                     "ENERGY",
                     "CHEMISTRY",
                     "EDUCATION",
                     "GREEN & SUSTAINABLE SCIENCE",
                     "TELECOMMUNICATIONS",
                     "MATERIALS SCIENCE",
                     "INSTRUMENTATION",
                     "OPTICS",
                     "BIOCHEMICAL SCIENCE",
                     "AGRICULTURE",
                     "ARCHITECTURE",
                     "PHYSICS",
                     "ENVIRONMENTAL SCIENCES",
                     "BIOLOGICAL SCIENCES",
                     "BIOLOGICAL SCIENCES",
                     "IMAGING SCIENCE")

  subject_2 <- subject
  i <- 1
  # Loop to replace subjects
    for(i in 1:length(pattern_x)) {
      # Replace pattern
        subject_2 <- subject_2 %>% mutate_all(funs(str_replace(., paste(pattern_x[i], ".*", sep = ""), replacement_x[i])))
    }
  
  # Remove any empty spaces
    subject_2 <- subject_2 %>% mutate(across(where(is.character), str_trim))
  
# Remove duplicate subjects in each row
    i <- 2
  for(i in 1:nrow(subject_2)) {
    subject_2[i, duplicated(c(subject_2[i,]))] <- NA
  }

# Merge strings back
  subject_3 <- subject_2 %>% unite(subject_2, a:g, sep = "; ") # From tidy r package
  
# Delete NAs
  subject_3$New <- gsub("; NA", "", subject_3$subject_2)

# Attach to M data frame
  M$Subject <- subject_3$New
  
# Subject growth
  #cdf = TRUE means cumulative growth
  subjects <- KeywordGrowth(M, Tag = "Subject", sep = ";", top = 12, cdf = TRUE)

# Convert country labels to sentence case
  names(subjects)[c(2:ncol(subjects))] <- to_any_case(names(subjects)[c(2:ncol(subjects))], case = "sentence")
  
# Plot area chart by country
  subjects_long <- melt(subjects, id='Year')
  
  # Calculate percentage change
    subjects_long <- subjects_long %>% group_by(variable) %>% mutate(pct_change = (value/lag(value)-1) * 100)
    
  # Subset to year 2021 only
    subjects_long_sub <- subjects_long[subjects_long$Year == 2021, ]
  
  # Display total sum of subjects
    datatable(subjects_long_sub[, c(2:3)])
```

```
 # Cumulative growth & yearly growth
    ggplot(subset(subjects_long, Year > 2010 & Year < 2021), aes(Year,value, 
                                   group=variable, 
                                   fill=variable))+
      geom_area(alpha=0.8, color='darkgrey', size = 0.1) +
      ggtitle("Cumulative growth")+
      labs(x="Publication year", y='Cumulative Web of Science records \n for open electronics boards', fill = "")+
      #scale_fill_brewer(palette="Dark1")+
      scale_fill_viridis(discrete = TRUE, option = "E")+
      theme_classic()+
      scale_x_continuous(breaks=seq(2006, 2020, 2))
```

```
    ggsave(paste(wd, "Subjects_CumulativeGrowthByYear.pdf", sep = ""), width = 6.5, height = 4)


# Horizontal bar chart
# Calculate sum of all subjects
  subjects_sum <- subjects_long_sub[,-4]
  subjects_sum$percentage <- round(subjects_sum$value/sum(subjects_sum$value)*100, 1)


# Horizontal bar plot using ggplot
  ggplot(subjects_sum, aes(x=variable, y=percentage)) +
    geom_bar(stat="identity") +
    coord_flip()+
    theme_classic()+
    theme(axis.text=element_text(size=14, color = "black"),
        axis.title=element_text(size=14),
        axis.line=element_blank())+
    geom_text(aes(label = paste0(percentage)), colour = "white",
            position = position_stack(vjust = 0.5), size = 2.5)+
            # vjust=0.5, hjust=-0.5,position = position_dodge(0.9), size = 4
    ylab("Overall share (%)")+
    xlab("Subject areas for open \n electronics boards WoS records")
```

```
  # Save plot
    ggsave(paste(wd, "Subjects_BarChart.pdf", sep = ""), width = 6.5, height = 4)
    ggsave(paste(wd, "Subjects_BarChart.svg", sep = ""), width = 6.5, height = 4)


#
```

# Display data

```
datatable(top_countries_long, editable = 'cell', filter = 'top')
```

# 8. Network analysis

## By keyword

```
# Author network
A <- cocMatrix(M, Field = "WC", sep = ";")
sort(Matrix::colSums(A), decreasing = TRUE)[1:10]
```

```
##             ENGINEERING, ELECTRICAL & ELECTRONIC               COMPUTER SCIENCE, THEORY & METHODS 
##                                             4765                                             2035 
##                               TELECOMMUNICATIONS            COMPUTER SCIENCE, INFORMATION SYSTEMS 
##                                             1665                                             1327 
##        COMPUTER SCIENCE, ARTIFICIAL INTELLIGENCE                     AUTOMATION & CONTROL SYSTEMS 
##                                             1132                                              895 
## COMPUTER SCIENCE, INTERDISCIPLINARY APPLICATIONS                   ENGINEERING, MULTIDISCIPLINARY 
##                                              813                                              631 
##                    INSTRUMENTS & INSTRUMENTATION        COMPUTER SCIENCE, HARDWARE & ARCHITECTURE 
##                                              615                                              496
```

```
# Author keyword network
A <- cocMatrix(M, Field = "DE", sep = ";")
sort(Matrix::colSums(A), decreasing = TRUE)[1:30]
```

```
##                  ARDUINO             RASPBERRY PI       INTERNET OF THINGS                      IOT 
##                     2039                     1436                      559                      523 
##              ARDUINO UNO                  SENSORS          MICROCONTROLLER         IMAGE PROCESSING 
##                      270                      265                      246                      171 
## INTERNET OF THINGS (IOT)                  ANDROID                   ZIGBEE                BLUETOOTH 
##                      166                      159                      131                      130 
##                      GSM          EMBEDDED SYSTEM  WIRELESS SENSOR NETWORK                      GPS 
##                      121                      120                      116                      115 
##                   SENSOR                   PYTHON         MACHINE LEARNING                     RFID 
##                      115                      114                      111                      110 
##                 SECURITY               AUTOMATION         EMBEDDED SYSTEMS            DEEP LEARNING 
##                      108                      108                      104                      101 
##          COMPUTER VISION                  LABVIEW               MONITORING  ARDUINO MICROCONTROLLER 
##                      101                      101                      101                       99 
##        ULTRASONIC SENSOR                   OPENCV 
##                       93                       88
```

```
# A classical keyword co-occurrences network
NetMatrix <- biblioNetwork(M, analysis = "co-occurrences", network = "keywords", sep = ";")
netstat <- networkStat(NetMatrix)

# Plot the network
net=networkPlot(NetMatrix, normalize="association", weighted=T, n = 50, Title = "Keyword Co-occurrences", type = "mds", size=T,edgesize = 5,labelsize=0.7)
```

## By country

```
# Create a country collaboration network
M <- metaTagExtraction(M, Field = "WC", sep = ";")
NetMatrix <- biblioNetwork(M, analysis = "collaboration", network = "countries", sep = ";")

# Plot the network

#pdf(paste(wd, "Network_Country.pdf", sep = ""), width = 15, height = 11)
svg(paste(wd, "Network_Country.svg", sep = ""), width = 10, height = 7)

#net=networkPlot(NetMatrix, n = dim(NetMatrix)[1], Title = "Country Collaboration", type = "fruchterman", #size=TRUE, remove.multiple=FALSE,labelsize=0.7,cluster="none")

#net=networkPlot(NetMatrix, n = 20, Title = "Country Collaboration", type = "fruchterman", size=TRUE, #remove.multiple=FALSE,labelsize=0.7,cluster="none")

set.seed(10)

net=networkPlot(NetMatrix, 
              n = 29, 
              Title = "Country Collaboration", 
              type = "fruchterman", 
              size=TRUE,
              remove.multiple=FALSE, 
              halo = TRUE, labelsize = 1.3, 
              curved = 0, 
              edges.min = 5, 
              remove.isolates = TRUE)

dev.off()
```

```
## png 
##   2
```
