# Supplementary material for: Open Hardware in Science: The Benefits of Open Electronics
Source: Integr Comp Biol. 2022 May 20;62(4):1061–75. doi: 10.1093/icb/icac043 (PMC9617215; doi:10.1093/icb/icac043)
Supplement: icac043_Supplemental_Files [file icac043_supplemental_files.zip › icb-2022-0062-File010.zip!/File_S2_SuppInfo/FullTextMining.html]

Full text mining across all PLOS journals for open electronics boards listed in Figure 3


# Full text mining across all PLOS journals for open electronics boards listed in Figure 3

#### Michael Oellermann

#### 21 July, 2021

# 1. Load libraries

```
library(fulltext)
library(pubchunks)
library(data.table)
library(rplos)
library(plyr)
library(dplyr)
library(ggplot2)
library(DT)
```

# 2. Search database

```
###################
# Search term copied from the search mask field under https://journals.plos.org/plosone/search
# ((((((((((everything:Arduino) OR everything:"Raspberry Pi") OR everything:"Asus Tinker Board") OR everything:"BeagleBone Black") OR everything:"Adafruit feather HUZZAH") OR everything:"Nvidia Jetson Nano") OR everything:"Odroid C4") OR everything:PocketBeagle) OR everything:"Raspberry Pi") OR everything:"Red Pitaya") OR everything:"UDOO Bolt"

# Search term for later filtering via grep
search_term <- "Arduino|Asus Tinker Board|BeagleBone Black|Adafruit feather HUZZAH|Nvidia Jetson Nano|Odroid C4|PocketBeagle|Raspberry Pi|Red Pitaya|UDOO Bolt"

# Search query
# Plos options look up here ?rplos::searchplos()
  plos_opt <- list(fl=c('id',
                        'author', 
                        'journal', 
                        "publication_date",
                        "subject"),
                   fq=list('doc_type:full',
                           "article_type:\"research article\""),
                   byfield='subject') 

# Search command 
  # (modify to 'everything:"Raspberry Pi"~15' to check words less than 15 words apart)
  q_result <- ft_search(query = '((((((((((everything:Arduino) OR everything:"Raspberry Pi") OR everything:"Asus Tinker Board") OR everything:"BeagleBone Black") OR everything:"Adafruit feather HUZZAH") OR everything:"Nvidia Jetson Nano") OR everything:"Odroid C4") OR everything:PocketBeagle) OR everything:"Raspberry Pi") OR everything:"Red Pitaya") OR everything:"UDOO Bolt"', 
                    from = 'plos', 
                    limit = 1000, 
                    plosopts = plos_opt)
```

# 3. Get full text

```
# Fetch article doi
  q_result_dois <- q_result$plos$data$id

# Get full texts
  q_result_full <- ft_get(q_result_dois, progress = TRUE)

  
# Pull various sections of each article.
  q_result_sections <- ft_collect(q_result_full)
  
# Pull out doi, title, abstract and body
  q_result_body <- pub_chunks(q_result_sections, 
                              sections = c("doi",
                                           "title",
                                           "body"))
  
  # Extract doi, title, abstract and body
    q_result_abstract <- pub_chunks(q_result_sections, 
                              sections = c("doi", 
                                           "abstract"))

# Convert results list to data frame
  q_result_body_df <- rbindlist(q_result_body$plos, fill = TRUE)
  q_result_abstract_df <- rbindlist(q_result_abstract$plos, fill = TRUE)
  
# Extract distinct entries for abstracts
  q_result_abstract_df <- q_result_abstract_df %>% distinct(doi, .keep_all = TRUE)
  
# Merge main text and abstract data frames
  q_result_df <- merge(q_result_body_df, q_result_abstract_df, by = "doi", all.x = TRUE)
  
# Remove last columns
  q_result_df <- q_result_df[, -c(4,6)]
  
# Remove old variables
  rm("q_result_abstract", "q_result_body", "q_result_abstract_df", "q_result_body_df", "q_result_dois")
```

# 4. Search for terms in article sections

```
# Search for term in abstract of text
  found_abstract <- ddply(q_result_df, .(doi), summarize,
        term_abstract = grep(search_term, abstract, value = TRUE))
  
# Search for term in main body of text
  found_body <- ddply(q_result_df, .(doi), summarize,
        paragraph_index = grep(search_term, body),
        term_body = grep(search_term, body, value = TRUE))
  
# Delete multiple matches in abstracts or main text
  found_abstract <- found_abstract %>% distinct(doi, .keep_all = TRUE)
  found_body <- found_body %>% distinct(doi, .keep_all = TRUE)

# Merge both results for abstracts and main text
  found_all <- merge(found_body, found_abstract, by = "doi", all = TRUE)
```

# 5. Identify subject category

```
# Pull out subject category from Plos search
  q_result_subject <- pub_chunks(q_result_sections, 
                              sections = c("doi",
                                           "categories"))

# Convert list to data frame
  q_result_subject_df <- rbindlist(q_result_subject$plos, fill = TRUE)
  
# Extract most frequent subject category for each article
  i <- 1
  # Empty vectors to save top terms
    top_cat_1 <- c()
    top_cat_2 <- c()
    top_cat_3 <- c()
  
  # Extract unique dois
    unique_dois <- unique(q_result_subject_df$doi)
    
    # Remove empty doi entries
      unique_dois <- na.omit(unique_dois)
  
  # Loop over articles and extract top category
    for(i in 1:length(unique_dois)){
      # subset by doi
        doi_group <- subset(q_result_subject_df, doi == unique_dois[i])
      # Create sorted frequency term table
        term_table <- sort(table(doi_group$categories), decreasing = TRUE)
      # Select most frequent and first listed term
        top_cat_1 <- c(top_cat_1, names(term_table)[1])
        top_cat_2 <- c(top_cat_2, names(term_table)[2])
        top_cat_3 <- c(top_cat_3, names(term_table)[3])
        #print(names(term_table)[1])
    }
    
    # Join terms into one data frame
      top_cat <- data.frame("doi" = unique_dois, top_cat_1, top_cat_2, top_cat_3)
    
# Merge search results with category
  found_all <- merge(found_all, top_cat, by = "doi")
  
# Add search term column
  found_all$search_term <- search_term
  # move term column to first position
    found_all <- found_all[,c(8, 1:7)]
```

# 6. Display data table

```
datatable(head(found_all, 100), editable = 'cell', filter = 'top')
```

# 7. Save results

```
# Save full texts
  saveRDS(q_result_full, paste("Fulltext_OpenElectronicsBoards.rds", sep = ""))

# Save results data frame
  write.csv(found_all, paste("Fulltext_OpenElectronicsBoards.csv", sep = ""))
```
