# Supplementary material for: Open Hardware in Science: The Benefits of Open Electronics
Source: Integr Comp Biol. 2022 May 20;62(4):1061–75. doi: 10.1093/icb/icac043 (PMC9617215; doi:10.1093/icb/icac043)
Supplement: icac043_Supplemental_Files [file icac043_supplemental_files.zip › icb-2022-0062-File010.zip!/File_S2_SuppInfo/Plot_SearchResults.html]

Plot open electronics search results


# Plot open electronics search results

#### Michael Oellermann

#### 21 July, 2021

# 1. Load libraries

```
library(ggplot2)
library(dplyr)
library(plyr)
library(reshape2)
library(data.table)
```

# 2. Load data

```
oe_results <- read.csv("Fulltext_OpenElectronicsBoards.csv")
```

# 3. Prepare data

```
# Remove row index column
  oe_results <- oe_results[, -1]

# Create frequency table for occurrences by subject category
  search_hit_freq <- ddply(oe_results, .(top_cat_1), summarize,
        term_freq_body = sum(!is.na(term_body)),
        term_freq_abstract = sum(!is.na(term_abstract)))

# Convert to long format
  search_hit_freq <- melt(search_hit_freq, id.vars=c("top_cat_1"))
```

```
## Warning in melt(search_hit_freq, id.vars = c("top_cat_1")): The melt generic
## in data.table has been passed a data.frame and will attempt to redirect to the
## relevant reshape2 method; please note that reshape2 is deprecated, and this
## redirection is now deprecated as well. To continue using melt methods from
## reshape2 while both libraries are attached, e.g. melt.list, you can prepend
## the namespace like reshape2::melt(search_hit_freq). In the next version, this
## warning will become an error.
```

```
# re-order by count
  search_hit_freq <- arrange(search_hit_freq, desc(value)) # arrange function from dplyr package
```

# 4. Plot data

```
## Total search counts
# Compare search term occurence frequency in abstract versus main text
  ggplot(search_hit_freq, aes(x = reorder(top_cat_1,(value)), y = value, fill = variable))+
    geom_bar(stat = "identity")+
    coord_flip() +
    ggtitle("Plos search hits for open electronics boards:\nAbstract versus main text") +
    #facet_wrap(~search_term)+
    ylab("Found OE terms in abstracts vs full text")+
    xlab("Plos categories")+
    theme_classic()
```

```
# save plot
  ggsave("SearchCounts_AbstractVSFullText.pdf")
```

```
## Saving 7 x 5 in image
```

```
## Ratio of full text versus abstract
  # Calculate ratio
    # Convert to wide
      search_hit_freq_wide <- reshape(search_hit_freq, idvar = c("top_cat_1"),
                                         timevar = "variable",
                                           direction = "wide")
    # Calculate ratio
      search_hit_freq_wide$ratioAbst_full <- search_hit_freq_wide$value.term_freq_abstract/ search_hit_freq_wide$value.term_freq_body * 100
        # Round ratios
          search_hit_freq_wide$ratioAbst_full <- round(search_hit_freq_wide$ratioAbst_full, 1)
        # Sort ratios
          search_hit_freq_wide <- arrange(search_hit_freq_wide, desc(value.term_freq_body))
      
    # Calculate total number of detected articles wit OE terms
      oehits_total <- sum(search_hit_freq_wide$value.term_freq_body)
      
  # Plot ratio of categories 
      # Subset data set to categories that have at least 4 search hits
        ratio_df <- search_hit_freq_wide %>% filter(value.term_freq_body > 3)
        
      # Plot ratios
      ggplot(ratio_df, aes(x = reorder(top_cat_1,(ratioAbst_full)), y = ratioAbst_full))+
      geom_bar(stat = "identity")+
      coord_flip() +
      ggtitle(paste("Plos search hits for open electronics boards:\nAbstract versus main text", "\nfound in ", oehits_total, " articles", sep = "")) +
      ylab("Abstracts containing OE terms relative to full text (%)")+
      xlab("Plos categories")+
      theme_classic()
```

```
# save plot
  ggsave("SearchCounts_AbstractVSFullText_ratio.pdf")
```

```
## Saving 7 x 5 in image
```

# 5. Save data

```
write.csv(search_hit_freq_wide, "OETerms_Abstract_vs_Fulltext.csv")
```
