# Supplementary material for: Open Hardware in Science: The Benefits of Open Electronics
Source: Integr Comp Biol. 2022 May 20;62(4):1061–75. doi: 10.1093/icb/icac043 (PMC9617215; doi:10.1093/icb/icac043)
Supplement: icac043_Supplemental_Files [file icac043_supplemental_files.zip › icb-2022-0062-File010.zip!/File_S2_SuppInfo/SearchCounts_AbstractVSFullText.pdf]

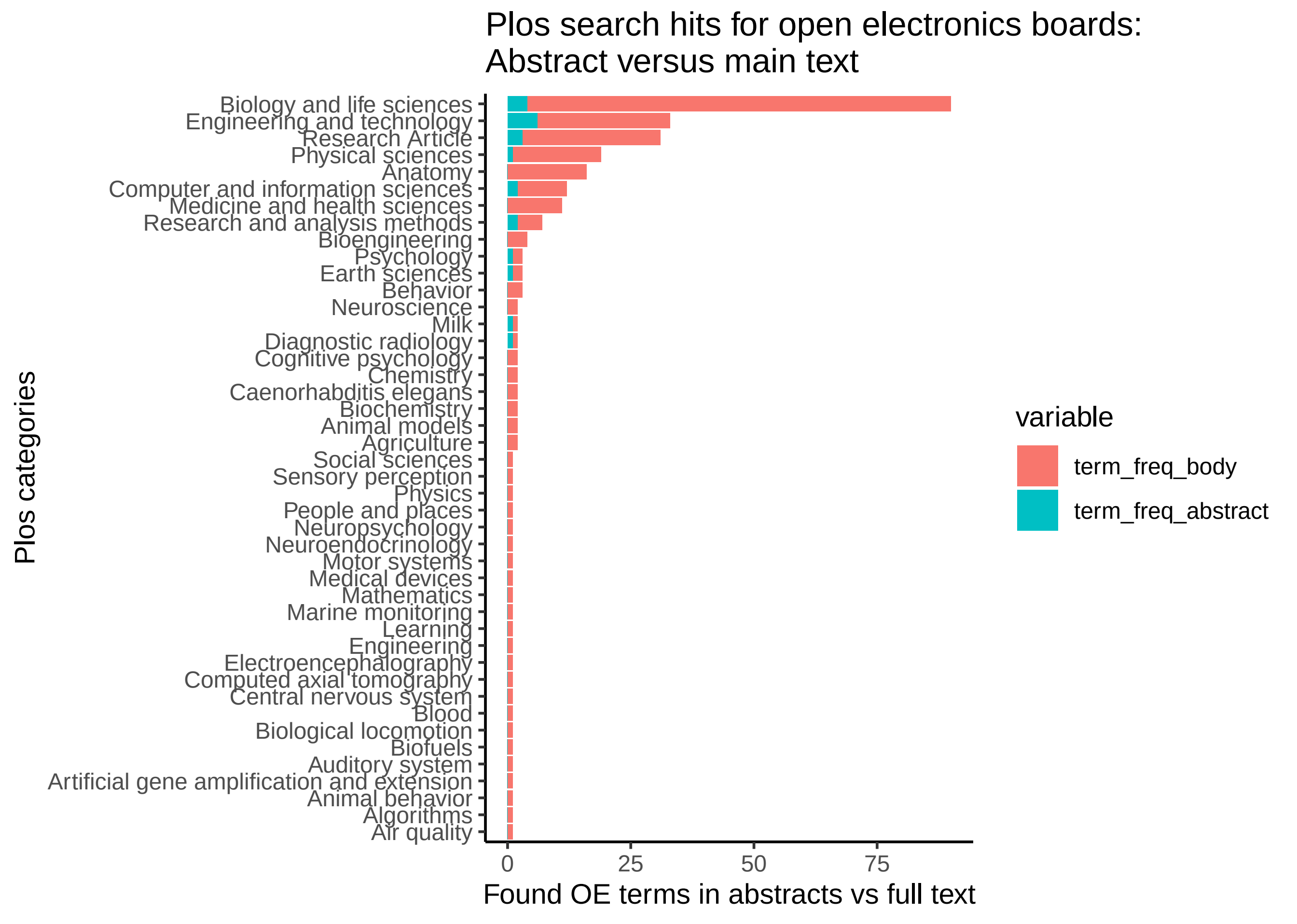

Plos search hits for open electronics boards:
Abstract versus main text
Biology and life sciences
Engineering and technology
Research Article
Physical sciences
Anatomy
Computer and information sciences
Medicine and health sciences
Research and analysis methods
Bioengineering
Psychology
Earth sciences
Behavior
Neuroscience
Milk
Diagnostic radiology
Cognitive psychology
Chemistry
Caenorhabditis elegans
Biochemistry
Animal models
Agriculture
Social sciences
Sensory perception
Physics
People and places
Neuropsychology
Neuroendocrinology
Motor systems
Medical devices
Mathematics
Marine monitoring
Learning
Engineering
Electroencephalography
Computed axial tomography
Central nervous system
Blood
Biological locomotion
Biofuels
Auditory system
Artificial gene amplification and extension
Animal behavior
Algorithms
Air quality
Plos categories
0
25
50
75
Found OE terms in abstracts vs full text
variable
term_freq_body
term_freq_abstract
