# Supplementary material for: Open Hardware in Science: The Benefits of Open Electronics
Source: Integr Comp Biol. 2022 May 20;62(4):1061–75. doi: 10.1093/icb/icac043 (PMC9617215; doi:10.1093/icb/icac043)
Supplement: icac043_Supplemental_Files [file icac043_supplemental_files.zip › icb-2022-0062-File010.zip!/File_S2_SuppInfo/SearchCounts_AbstractVSFullText_ratio.pdf]

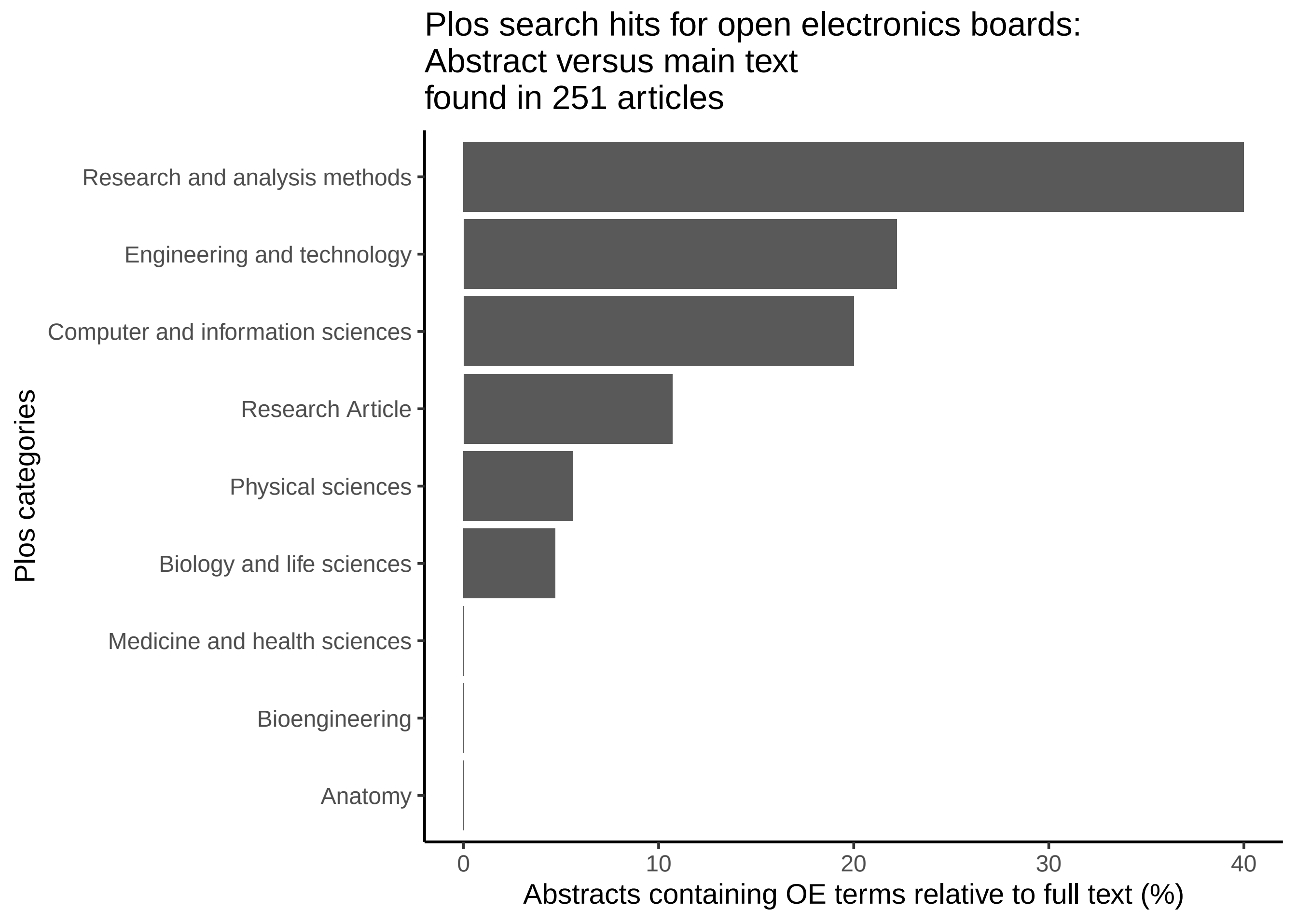
Plos search hits for open electronics boards:
Abstract versus main text
found in 251 articles
Research and analysis methods
Engineering and technology
Computer and information sciences
Research Article
Physical sciences
Biology and life sciences
Medicine and health sciences
Bioengineering
Anatomy
Plos categories
0
10
20
30
40
Abstracts containing OE terms relative to full text (%)
